# Susceptibility to hormone-mediated cancer is reflected by different tick rates of the epithelial and general epigenetic clock

**DOI:** 10.1186/s13059-022-02603-3

**Published:** 2022-02-22

**Authors:** James E. Barrett, Chiara Herzog, Yoo-Na Kim, Thomas E. Bartlett, Allison Jones, Iona Evans, David Cibula, Michal Zikan, Line Bjørge, Nadia Harbeck, Nicoletta Colombo, Sacha J. Howell, Angelique Flöter Rådestad, Kristina Gemzell-Danielsson, Martin Widschwendter

**Affiliations:** 1 European Translational Oncology Prevention and Screening (EUTOPS) Institute, Milser Str. 10, 6060 Hall in Tirol, Austria; 2 Research Institute for Biomedical Aging Research, Universität Innsbruck, 6020 Innsbruck, Austria; 3 Department of Statistical Science, University College London, WC1E 7HB, London, UK; 4 Department of Women’s Cancer, UCL EGA Institute for Women’s Health, University College London, Medical School Building, Room 340, 74 Huntley Street, WC1E 6AU London, UK; 5 Gynaecologic Oncology Center, Department of Obstetrics and Gynecology, First Faculty of Medicine, Charles University in Prague, General University Hospital in Prague, Prague, Czech Republic; 6 Department of Gynecology and Obstetrics, Charles University in Prague, First Faculty of Medicine and University Hospital Bulovka, Prague, Czech Republic; 7 Department of Obstetrics and Gynaecology, Haukeland University Hospital, Bergen, Norway; 8 Centre for Cancer Biomarkers CCBIO, Department of Clinical Science, University of Bergen, Bergen, Norway; 9 Breast Center, Department of Obstetrics and Gynecology, University of Munich (LMU), Munich, Germany; 10 Istituto Europeo di Oncologia IRCCS, Milan, Italy; 11 University of Milano-Bicocca, Milan, Italy; 12 Breast Biology Group, Manchester Breast Centre, Division of Cancer Sciences, Faculty of Biology, Medicine and Health, University of Manchester, Manchester, UK; 13 Department of Women’s and Children’s Health, Karolinska Institutet and Karolinska University Hospital, Stockholm, Sweden

## Abstract

**Background:**

A variety of epigenetic clocks utilizing DNA methylation changes have been developed; these clocks are either tissue-independent or designed to predict chronological age based on blood or saliva samples. Whether discordant tick rates between tissue-specific and general epigenetic clocks play a role in health and disease has not yet been explored.

**Results:**

Here we analyze 1941 cervical cytology samples, which contain a mixture of hormone-sensitive cervical epithelial cells and immune cells, and develop the WID general clock (Women’s IDentification of risk), an epigenetic clock that is shared by epithelial and immune cells and optimized for cervical samples. We then develop the WID epithelial clock and WID immune clock, which define epithelial- and immune-specific clocks, respectively. We find that the WID-relative-epithelial-age (WID-REA), defined as the difference between the epithelial and general clocks, is significantly reduced in cervical samples from pre-menopausal women with breast cancer (OR 2.7, 95% CI 1.28-5.72). We find the same effect in normal breast tissue samples from pre-menopausal women at high risk of breast cancer and show that potential risk reducing anti-progesterone drugs can reverse this. In post-menopausal women, this directionality is reversed. Hormone replacement therapy consistently leads to a significantly lower WID-REA in cancer-free women, but not in post-menopausal women with breast or ovarian cancer.

**Conclusions:**

Our findings imply that there are multiple epigenetic clocks, many of which are tissue-specific, and that the differential tick rate between these clocks may be an informative surrogate measure of disease risk.

**Supplementary Information:**

The online version contains supplementary material available at 10.1186/s13059-022-02603-3.

## Background

Age-associated DNA methylation alterations occur at numerous CpG sites across the human genome and form the basis of epigenetic clocks. Specific sites like those regions which, in stem cells, are occupied by the polycomb-repressor complex 2 become preferentially methylated with increasing age [1–3]. By taking a combination of multiple age-associated differentially methylated positions (aDMPs), these clocks offer remarkably accurate predictions of chronological age [4–8]. Epigenetic age acceleration, defined as the difference between epigenetic and chronological age, has been associated with cancer risk, mortality, as well as several other diseases [4, 9–15].

Many age-related DNA methylation changes are common across most tissue types [16], and one of the earliest epigenetic clocks was designed as a pan-tissue clock using samples from multiple tissues [4]. Other aDMPs are highly tissue specific [17–19], and alternative clocks have been developed using blood [5, 6] and saliva samples [7].

There are suggestions that the tick rate of epigenetic clocks allows for the prediction of disease and mortality risk [20]. Both the number of cell divisions and replication-independent factors determine the pace of epigenetic clocks; hence in order to utilize an epigenetic clock as a surrogate marker to monitor disease risk, the cell-type and conditions which impact on this cell type have to be considered. This is of particular importance for multifactorial diseases like cancer where hormonal exposure is crucially involved in cancer development and cell division is limited to cells that are hormone receptor positive.

Here we analyzed cervical liquid-based cytology samples from 1941 women which, due to the heterogeneous nature of the sample, provide direct access to both hormone-sensitive epithelial cells from the cervix and non-epithelial cells. We have developed epigenetic clocks which reflect the epithelial and immune specific age as well as a general tissue-independent clock. We aimed to assess whether these clocks, and any discordance between them, are associated with women’s cancers that do not have their origin at the cervix, and how this depends on menopausal status and hormone exposure.

## Results

### Development of a tissue-independent epigenetic clock

**Fig. 1:**
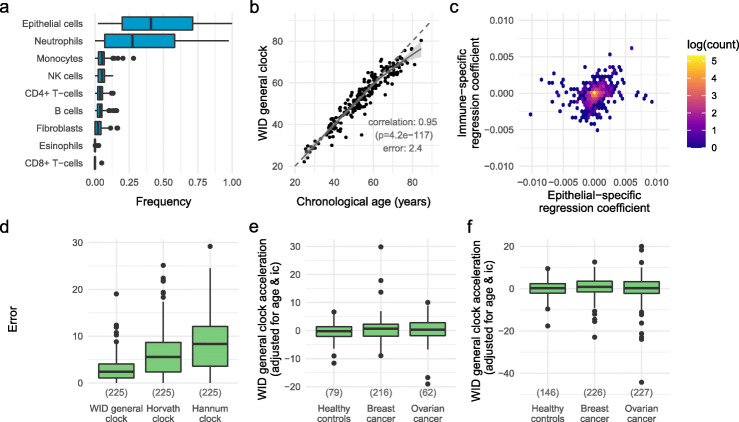
Overview of the WID general clock. **a** Cell-type composition of cervical samples in the training set. **b** WID general clock versus chronological age in control samples from Validation set 1. **c** Distribution of regression coefficients of either epithelial- or immune-specific aDMPs (*x* or *y*-axis, respectively) in the 759 age index CpGs. **d** Error (absolute difference between predicted and chronological age) in the WID general clock compared to existing clocks in cervical samples. **e**, **f** Age- and ic-adjusted age-acceleration (Δ WID-general clock and chronological age) in pre- (**e**) and post-menopausal (**f**) samples from healthy cancer-free women and women with breast or ovarian cancer. *P* values for correlations were calculated using Pearson’s product-moment correlation coefficient

We analyzed cervical cytology samples from 869 healthy women (593 from the general population and 276 from women attending hospital for benign women-specific conditions; Additional file 1: Fig. S1, Supplementary Table S1) as part of our training dataset. Samples were processed on the Illumina EPIC array, which covers approximately 850,000 CpG sites. We used the EpiDISH algorithm to infer the relative proportion of epithelial cells, fibroblasts, and seven subtypes of immune cells in each sample. Cervical samples are highly heterogeneous, consisting of a mixture of cervical, epithelial, and immune cells in which the immune cell (ic) proportion follows a roughly uniform distribution (Fig. 1a).

We used lasso regression to develop an epigenetic clock termed the WID general clock (Women’s IDentification of risk). We trained the clock to predict chronological age using beta values from 776,725 CpGs in our training dataset. The final clock consisted of a linear combination of 759 CpGs. We evaluated the performance of the clock on an independent validation dataset (Additional file 1: Supplementary Table S2) of 225 cervical cytology samples from healthy women (Fig. 1b). The error (defined as the median absolute difference between the true and predicted age) was 2.4 years and the Pearson correlation was 0.95 (*p* < 10^−16^). We observed a slight underestimation of age in older individuals, consistent with previous reports in the literature [21].

In order to determine whether the CpGs used in the clock were specific to epithelial or immune cells, or shared across both, we used a linear model to estimate epithelial- and immune-specific age-associated changes. The majority of changes were shared across both cell types (Fig. 1c). The clock accuracy was largely independent of cell-type composition. In samples with an immune cell (ic) proportion < 0.5, the error was 2.61 years and the correlation was 0.95 (*p* < 10^−16^). In samples with ic ≥0.5, the error was 2.18 years and the correlation was 0.96 (*p* < 10^−16^). We found that in cervical samples the WID general clock offered substantially superior performance in comparison to the most frequently quoted clocks previously published by Horvath [4] and Hannum [5] (Fig. 1d, Additional file 1: Fig. S2a, b). This was expected as our clock was optimized for cervical cytology specimens. We evaluated the performance of all three clocks in fat, blood, muscle, colon, saliva, breast, skin, brain, buccal, and bone tissue samples and found that the WID general clock offers superior performance in all but buccal and bone tissues (Additional file 1: Fig. S2c).

### Age acceleration in breast, ovarian, and endometrial cancers

We observed that age-acceleration, defined as the difference between the WID general clock and chronological age, was strongly dependent on chronological age (Additional file 1: Fig. S2d) and largely independent of immune cell proportion (Additional file 1: Fig. S2e). Epidemiological factors and cancer also did not influence immune cell proportion (Additional file 1: Fig. S2f-h). Nonetheless, in all subsequent analyses of age-acceleration, we used residuals after regressing on age and ic using the 225 control samples from the validation set.

We next analyzed cervical cytology samples from 442 women with breast cancer and 289 with ovarian cancer (all collected at the time of diagnosis). These samples had a comparable cell type composition to the healthy control samples (Additional file 1: Fig. S2i). Neither in pre- (Fig. 1e) nor in post-menopausal (Fig. 1f) women did we observe a significant difference in age-acceleration (adjusted for age and ic) between sample types.

We next examined healthy controls and cancers with respect to hormone replacement therapy (HRT), oral contraceptive pill (OCP) use, menopause, age at menopause, age at menarche (previous studies found an association between age-acceleration and age at menarche in blood [22] and age at menopause [23]), smoking, and obesity but did not observe any significant associations (Additional file 1: Fig. S3).

### Development of tissue-specific epigenetic clocks

**Fig. 2:**
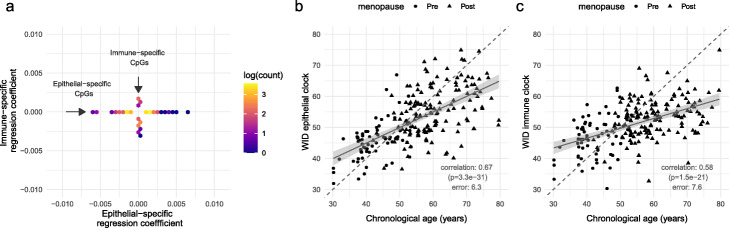
Description of the WID epithelial and immune clocks. **a** Distribution of the 168 epithelial- and 56 immune-specific aDMPs (*x* or *y*-axis, respectively) which were used to construct epithelial and immune clocks. **b** WID epithelial clock and **c** WID immune clock versus chronological age in cervical samples from healthy controls

The WID general clock was optimized for heterogeneous samples and as a consequence is largely based on CpGs that vary with age in both epithelial and immune cells. We hypothesized that the WID general clock defines a shared tick rate that is present across all cell types in cervical cytology samples. In order to develop tissue-specific clocks, we applied a linear model to each of the 776,725 CpGs in our training set and identified 168 epithelial-specific aDMPs (defined as CpGs with an absolute epithelial-specific coefficient > 10^−3^ and an absolute immune-specific coefficient < 10^−5^), and 56 immune-specific aDMPs (Fig. 2a).

We used ridge regression on these 168 epithelial- and 56 immune-specific aDMPs in the training dataset in order to derive an epithelial clock termed the WID epithelial clock, and an immune clock termed the WID immune clock. Both clocks are defined as a linear combination of beta-values and beta-proportion interaction terms (to account for any dependence on immune cell proportion). Both the epithelial and immune clocks had errors of 6.3 years (correlation 0.67, *p* < 10^−16^) and 7.6 years (correlation 0.58, *p* < 10^−16^) respectively, which was poorer performance than the tissue-independent clock (Fig. 2b, c).

Cervical epithelial cells are steroid hormone (i.e., estrogen and progesterone) sensitive; menopause (which occurs at around 50 years of age) leads to dramatically decreased levels of steroid hormones and we found a significant change in the general and epithelial clock after menopause (Additional file 1: Fig. S4a), suggesting that the clocks can be influenced by hormonal changes occurring at menopause.

The general, epithelial and immune clocks are significantly, albeit weakly, correlated with two mitotic clocks, the pcgtAge score based on promoter CpGs at polycomb group target genes [24], and an alternative mitotic clock model recently developed using “solo-WCGWs” [25], i.e. CpGs occurring in a WCGW sequence context that lose methylation as a result of incomplete methylation maintenance during cell division (Additional file 1: Fig. S4b-g). The weak correlation with mitotic clocks, despite excellent correlation with chronological age, implies that the WID clocks reflect age-associated epigenetic changes that are to a large extent independent of mitotic age.

### Relative epithelial age

The epithelial and immune clocks are derived from aDMPs that are specific to epithelial and immune cells respectively, but since they are simply a linear combination of beta-values, they define epigenetic clocks in any cell. We can therefore interpret each cell as possessing three separate epigenetic clocks (general, epithelial, and immune). The tick rate of each clock is cell type specific; the general clock serves as a good predictor of age across all cell types whereas the epithelial clock acts as a better predictor in epithelial cells and so forth.

**Fig. 3:**
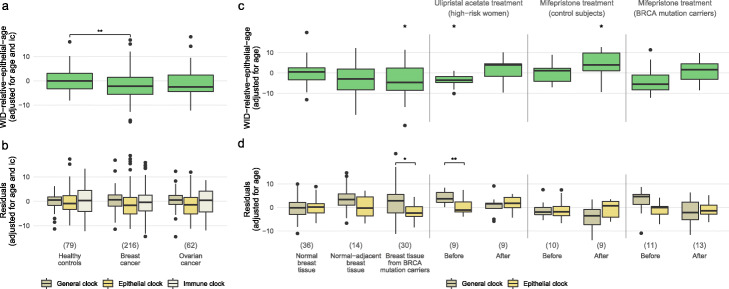
A discordance between the general and epithelial clock indicates breast cancer risk in cervical samples and tissue at risk in premenopausal women and can be rescued with progesterone antagonist treatment. **a**, **b** WID-relative-epithelial-age (WID-REA; difference between general and epithelial clock, **a**), and visualization of the general, epithelial, and immune clocks (after adjustment for age and ic, **b**) in cervical samples of pre-menopausal healthy controls and cancer cases in Validation set 1. **c**, **d** WID-REA, general, and epithelial clocks in breast tissue: normal breast tissue, normal-adjacent breast tissue samples from women with triple-negative breast cancer, normal breast tissue from BRCA mutation carriers, and samples before and after treatment with the potential breast cancer preventive drugs ulipristal acetate or mifepristone. Ulipristal acetate treatment was carried out in women at high risk of breast cancer (> 17% life-time breast cancer risk based on the Tyrer-Cuzick model). Mifepristone treatment was carried out either in healthy controls or BRCA mutation carriers. Samples before mifepristone treatment are a subset of the 36 normal breast tissue samples and 30 breast tissue samples from BRCA mutation carriers. *p value 0.05; **p value 0.01 in one-way ANOVA with Tukey’s post test (**a**, **b**). *p value 0.05 in Wilcoxon test (unpaired or paired, for top and bottom graph in **c** and **d**, respectively)

When we examined the general, epithelial, and immune clocks in pre-menopausal women, we observed a slightly increased general tick rate and a reduced epithelial tick rate in  those with breast and ovarian cancers although neither were significant (Fig. 3b). This motivated us to define the WID-relative-epithelial-age (WID-REA) as the difference between the epithelial and general clocks. The WID-REA was strongly dependent on age but not ic (Additional file 1: Fig. S5a, b). The WID-REA (adjusted for age and ic proportion) in pre-menopausal women with breast cancers was significantly lower in comparison to samples from healthy cancer-free controls (*p* = 0.013; Fig. 3a). Women in the lowest quartile of WID-REA index values have a breast cancer odds ratio of 2.7 (95% CI: 1.28–5.72) (Additional file 1: Supplementary Table S3). Interestingly, this effect appeared to be driven by estrogen and particularly progesterone receptor positive breast cancers (Additional file 1: Fig. S5e). A reduced WID-REA was also present in  women with pre-menopausal ovarian cancers but was not statistically significant.

We next evaluated the WID-REA in normal breast tissue samples from 36 healthy cancer-free pre-menopausal women, 14 normal-adjacent breast tissue samples from women with triple-negative breast cancer, and 30 *BRCA* mutation carriers (Additional file 1: Supplementary Table S4). We aimed to assess whether samples at high risk of breast cancer (i.e., normal breast tissue adjacent to breast cancer and normal breast from *BRCA* mutation carriers) had a reduced WID-REA, similar to that observed in cervical samples. We adjusted the WID-REA for age only because, unlike cervical samples, there is relatively little variability in the cell-type composition of breast tissue samples (Additional file 1: Fig. S5f). Consistent with our observations in cervical samples, we found an increased general tick rate and a decreased epithelial tick rate in the normal-adjacent and *BRCA* samples leading to a lower WID-REA (Fig. 3c, d).

Progesterone is a key player in the development of breast cancer [26–28]. Therefore, we assessed whether potential anti-progesterone breast cancer preventive drugs, ulipristal acetate and mifepristone [29], are able to reverse these discordant tick rates. We assessed the WID-REA in breast tissue samples from 9 healthy cancer-free women before and after 3 months of ulipristal acetate treatment. These women had a high risk of breast cancer (> 17% lifetime risk of developing breast cancer according to the Tyrer-Cuzick risk model [30]) which was reflected in a reduced WID-REA. After treatment, the discordance was completely reversed and the median WID-REA increased above zero (Fig. 3c, d).

We then analyzed 10 healthy women and 13 healthy *BRCA* mutation carriers before and after a three-month treatment with mifepristone. In healthy women, the median WID-REA significantly increased above zero, indicating a potential protective effect (Fig. 3c, d). In *BRCA* carriers, a negative WID-REA was increased to values comparable to healthy women.

### Hormone replacement therapy

**Fig. 4:**
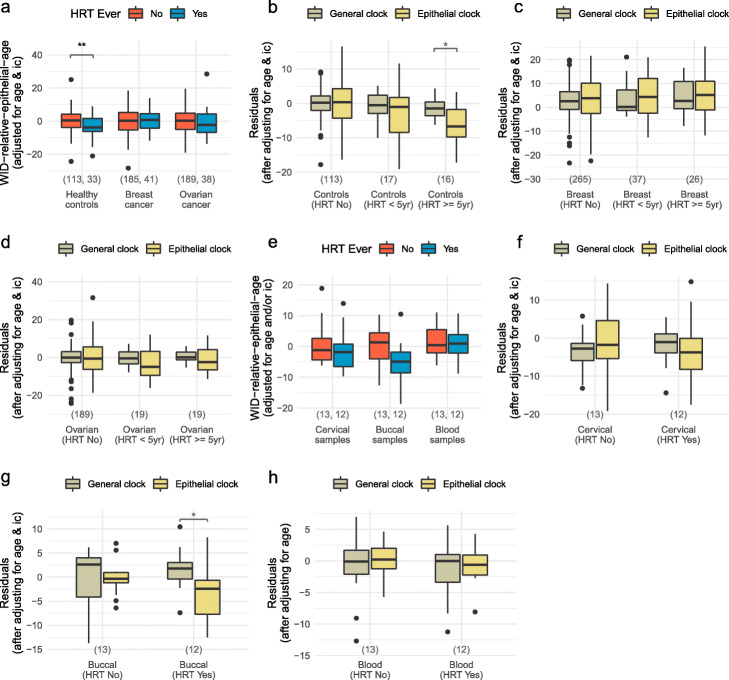
The WID relative epithelial age in post-menopausal women is influenced by hormone replacement therapy. **a** WID-REA with respect to hormone replacement therapy (HRT) in cervical samples from post-menopausal cancer-free controls and cancers from Validation set 1. The general and epithelial clocks versus HRT treatment duration in cervical samples post-menopausal **b** cancer-free controls, **c** breast, and **d** ovarian cancer from Validation set 1. **e** WID-REA with respect to HRT in matched cervical, buccal, and whole blood in post-menopausal samples from Validation set 2. The general and epithelial clocks in **f** cervical, **g** buccal, and **h** whole blood in post-menopausal samples from Validation set 2. *p 0.05; **p 0.01 in paired Wilcoxon tests

We next examined the WID-REA with respect to hormone replacement therapy (HRT). Post-menopausal cancer-free control women who were ever on HRT showed a significantly reduced WID-REA (*p* = 0.006; Fig. 4a). The reduced WID-REA in cancer-free controls increased with HRT duration, consistent with a retardation of the epithelial clock (relative to the general clock) that becomes more pronounced over time (Fig. 4b). Surprisingly, HRT had no effect at all on the WID-REA in post-menopausal women with breast or ovarian cancer (Fig. 4c, d). This was surprising and indicated that the meaning of a negative WID-REA was strongly dependent on menopausal status: in pre-menopausal women a negative WID-REA is associated with increased cancer risk, whereas in a post-menopausal context this directionality is reversed.

To validate this observation further, we analyzed cervical, buccal, and blood samples from an independent cohort of 116 healthy cancer-free women, of whom 13 were never and 12 were on HRT (Additional file 1: Supplementary Table S5). We observed a similar reduction of the WID-REA in post-menopausal women ever on HRT in cervical and buccal samples, although this did not reach significance (Fig. 4e). As expected, this effect was absent in blood samples, consistent with the fact that the epithelial clock was developed using epithelial-specific aDMPs. Examination of the general and epithelial clocks in these samples confirms the effect is driven by a slower epithelial tick rate (Fig. 4f–h).

Additionally, we analyzed the WID-REA with respect to OCP use, menopause, age at menopause, age at menarche, smoking, and obesity but did not observe any significant associations (Additional file 1: Fig. S6). We similarly defined the relative-immune-age as the difference between the immune and general clocks. We noted minor, but non-significant, associations with cancers but otherwise did not observe any associations with the above factors (Additional file 1: Fig. S7).

## Discussion

Here, we have leveraged the heterogeneous nature of smear samples from the uterine cervix in order to develop epithelial- and immune-specific epigenetic clocks, as well as a general clock that acts as an accurate predictor of age in both cell types.

Although in the past many epigenetic clocks have been developed, not one of these clocks has considered the relationship between cell subtype-specific (i.e., epithelial) and general epigenetic clocks and whether the discordance between different clocks, particularly when assessed in surrogate tissue, reflects disease risk. The difference in tick rates between the epithelial and general clocks, a quantity we termed the WID-relative-epithelial-age (WID-REA), captures an intrinsic cellular tension. When assessed in cervical smear samples it offers a highly informative measure of the cancer risk in non-cervical hormone-dependent tissues (i.e., particularly for breast cancer, by far the most common cancer) and is susceptible to hormonal modulation.

**Fig. 5:**
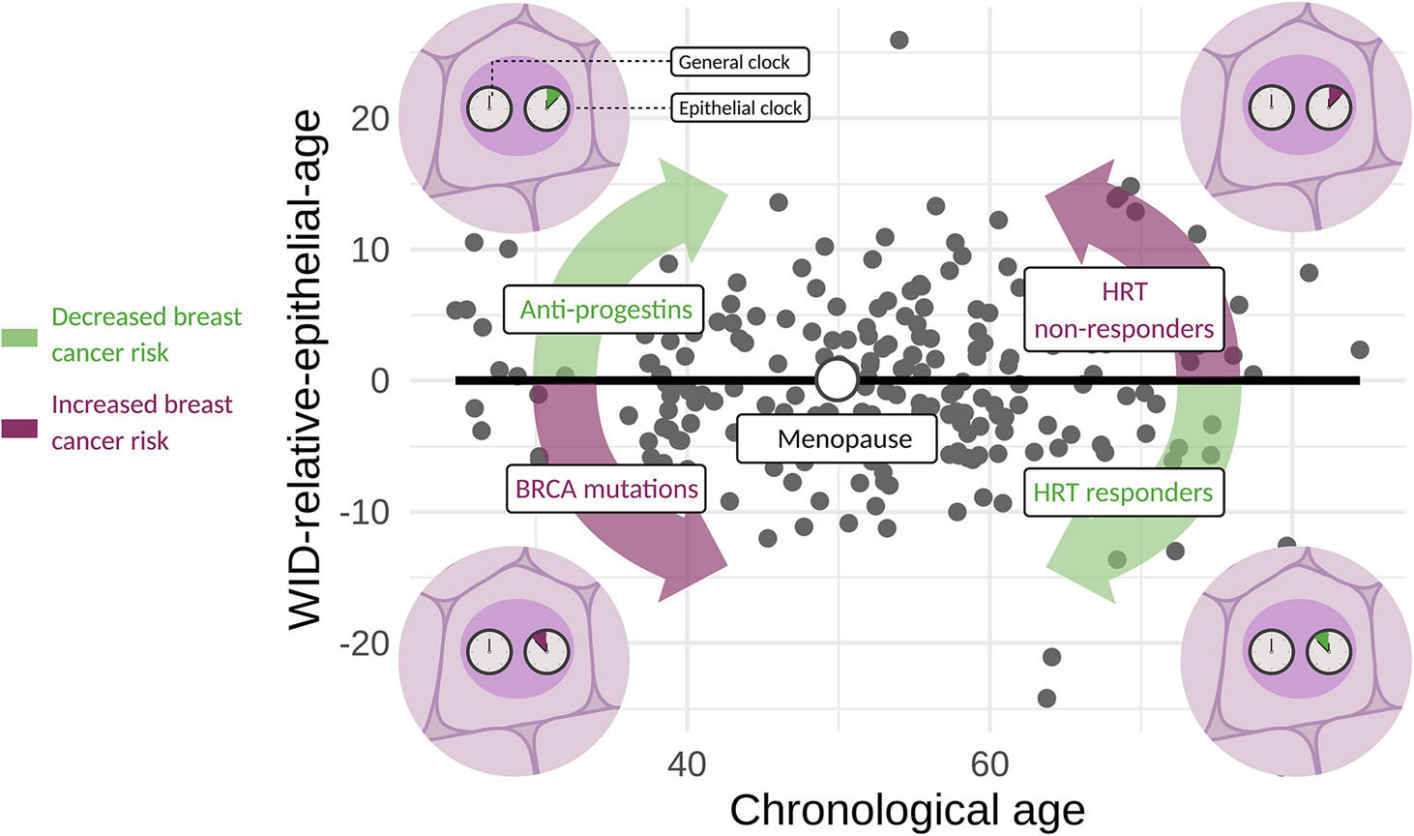
Menopause as a turning point for the WID-relative-epithelial-age: distinct impacts of hormonal exposure or genetic factors on the WID-relative-epithelial age before or after menopause. Before menopause, factors increasing breast cancer risk (e.g., *BRCA* mutations) result in a reduced WID-relative-epithelial-age, while anti-progestins (such as ulipristal acetate or mifepristone) *increase* the WID-relative-epithelial-age. After menopause, this pattern is switched: HRT responders exhibit a *reduced* WID-relative-epithelial-age, but this effect is not observed in HRT non-responders and may indicate an increased risk for breast cancer in these women. Therefore, an acceleration or deceleration of the WID-relative-age (age discordance) has different effects before and after menopause

The relationship between the WID-REA and cancer (i.e., breast cancer) risk is strongly dependent on menopausal status (Fig. 5). In pre-menopausal women, conditions which are associated with higher exposure to progesterone (e.g., a *BRCA* mutation) lead to a slower epithelial clock compared to the general clock and a higher breast cancer risk. The potential breast cancer risk reducing drugs ulipristal acetate and mifepristone rectify this discordance in breast tissue. In post-menopausal women, a HRT-mediated selective “anti-aging effect” of epithelial cells (i.e., reduced WID-REA) is prevalent in women with no cancer but absent in women with breast and ovarian cancer.

Overall, our data provide strong evidence that the WID-REA captures the concordance or discordance of the epithelial and general epigenetic clock. Importantly, the WID-REA in cervical samples acts as a systemic surrogate indicating how individual women respond to hormonal modulation and, depending on the menopausal status, how this is associated with cancer predisposition.

Although we have been able to demonstrate in sequential normal breast tissue samples that anti-progestins are able to rectify discordant tick rates, future research will assess (i) whether breast cancer risk reduction utilizing anti-progestins can be monitored using cervical samples and (ii) if individual post-menopausal women who decide to commence HRT, and do not demonstrate a reduction of the WID-REA after a certain time on HRT, should be advised against continuation of HRT and seek alternative measures. Cervical cytology samples are routinely collected as part of many national screening programs and self-collection kits allow women to collect samples at home. These samples therefore provide a unique and promising opportunity for monitoring cancer risk and response to HRT or preventive measures.

## Conclusions

Our findings imply that there are multiple epigenetic clocks, many of which are tissue-specific, and that the differential tick rate between these clocks may be an informative surrogate measure of disease risk.

## Methods

### Study design and epidemiological data acquisition

The study was conducted as part of a multi-center study involving several recruitment sites in five European countries (i.e., the UK, Czech Republic, Italy, Norway, and Germany). Women over the age of 18 years who had not undergone a previous hysterectomy, not received treatment (within 2 years of recruitment) for a non-gynecological cancer, were not pregnant or menstruating at the time of recruitment, and had not undergone a cervical smear in the last 12 weeks were eligible for this study. Women diagnosed with breast and ovarian cancer (case), or a non-malignant benign gynecological condition (control), were approached during outpatient hospital clinics, while women recruited as healthy volunteers from the general population (control) were approached via outreach campaigns, public engagement, and as part of cervical screening programs. After signing an informed consent, participants completed an epidemiological questionnaire as well as a feedback form after their participation. The study itself is a sub-study of the FORECEE (4C) Program, which has ethical approval from the UK Health Research Authority (REC 14/LO/1633) and other contributing centers.

Cervical samples were taken at collaborating hospitals and recruitment centers using the ThinPrep system (Hologic Inc., cat #70098-002), by rotating a Cervex brush (Rovers Medical Devices, cat #70671-001) for 5 times through 360° while in contact with the cervix. The sample vial was sealed and stored locally at room temperature. Buccal cells were collected using two Copan 4N6FLOQ Buccal Swabs (Copan Medical Diagnostics, cat #4504C) by firmly brushing the swab head 5–6 times against the buccal mucosa of each cheek. The swabs were re-capped and left to dry out at room temperature within the sampling tube which contains a drying desiccant. 2.5 mL of venous whole blood were collected in PAX gene blood DNA tubes (BD Biosciences #761165) and stored locally at 4 °C. All samples were shipped to University College London (UCL) at ambient temperature.

Biological samples were given an anonymous Participant ID Number, which was assigned to the person’s name in a securely stored link file. Following sample taking, an email survey was sent to participants, enabling them to feedback with respect to the recruitment process. Women with a current diagnosis of (a) primary breast cancer with poor prognosis features (Grade III and/or T2/3 and/or N1/2 and/or HR-ve) or (b) malignant invasive epithelial ovarian cancer, and recruited prior to receiving any systemic treatment (chemo- or antihormonal or Herceptin, etc.) or surgery or radiotherapy, were eligible as breast or ovarian cancer cases respectively. Controls were initially matched one-to-one with cases based on menopausal status, age (5 year age ranges where possible), and recruitment center/country. However, due to an imbalance in recruitment of cases and controls at some centers, a number of cases were matched on age and menopausal status alone.

### Breast tissue samples

Normal breast tissue samples (*n* = 36) included samples from 14 women who underwent cosmetic breast operations and 22 samples from a clinical trial called “The Effect of a Progesterone Receptor Modulator on Breast Tissue in Women With BRCA-1 and -2 Mutations - a Placebo Controlled RCT” (ClinicalTrials.gov Identifier: NCT01898312; regional ethical review board at the Karolinska Institutet permit 2009/144-31/4) consisting of healthy pre-menopausal women aged 18–43 years before treatment with mifepristone (*n* = 10) or placebo (*n* = 12). Normal-adjacent breast tissue samples were acquired from 14 women who had breast surgery due to a triple-negative breast cancer. Normal breast tissue samples from *BRCA* mutation carriers (*n* = 30) were obtained from risk-reducing surgeries (*n* = 14, 10 *BRCA1* and 4 *BRCA2*) and *BRCA* mutation carriers from the mifepristone clinical trial before treatment with placebo (*n* = 5) or mifepristone (*n* = 11). Ethical approval for collection of control cosmetic surgery samples and samples from *BRCA* mutation carrier risk-reducing surgeries was obtained from the NRES Committee East of England (reference number 15/EE/0192). The ulipristal acetate dataset consisted of breast tissue samples obtained as part of the Breast cancer anti-progestin study 1 (BC-APPS1 (2014MayCR003); regional ethical review board at Greater Manchester – South, Research Ethics Committee REC number 15/NW/0478) funded by Breast Cancer Now. The BC-APPS1 was a single-arm study designed to test the efficacy and safety of ulipristal acetate in breast cancer prevention and included 9 pre-menopausal women aged 38 years or below with a ≥ 1:6 lifetime risk of breast cancer (assessed by Tyrer-Cuzick v8). Breast tissue samples were obtained by vacuum-assisted biopsy at baseline and contralaterally 12 weeks following ulipristal acetate. All samples were collected fresh from theater and samples processed within 1 h of surgical excision. Fresh samples were frozen rapidly in Liquid Nitrogen and stored at −80 °C.

### Tissue samples for comparison of epigenetic clocks

Twelve datasets were downloaded from the GEO (Additional file 1: Supplementary Table S6) and the WID general clock, the Horvath clock, and the Hannum clock were computed for each sample. Only samples from healthy disease free individuals were considered. The breast tissue samples consisted of the 36 normal samples from our breast tissue validation set described above. The blood samples were a combination of the 116 blood samples from our validation set 2 and five other blood GEO datasets. The buccal samples were a combination of the 116 buccal samples from our validation set 2 and one additional GEO dataset.

### Sample processing and DNA extraction

When preparing for sample storage in the laboratory, cervical samples were poured into 50 mL Falcon tubes and left to sediment at room temperature for 2 h. One-milliliter wide bore tips were then used to transfer the enriched cellular sediment into a 2 mL vial. The cervical sediments were washed twice with PBS, lysed, and temporarily stored at − 20 °C ahead of extraction. The Copan 4N6FLOQ Buccal Swabs were cut and lysed sequentially in the same aliquot of lysis buffer prior to temporary storage at − 20 °C ahead of extraction. Whole blood samples were simply held transiently at − 20 °C until DNA extraction. DNA was extracted from cervical and buccal tissue lysates on a Hamilton Star liquid handling platform using the Nucleo-Mag Blood 200 μL kit (Macherey Nagel, cat #744501.4) with prior modifications for optimal lysis of cervical cell pellets and paired buccal swabs. DNA concentration and quality absorbance ratios were measured using Nanodrop-8000, Thermoscientific Inc. Extracted DNA was stored at − 80 °C until further analysis.

### DNA methylation array analysis

Cervical and buccal tissue DNA was normalized to 25 ng/μL and 500 ng total DNA was bisulfite modified using the EZ-96 DNA Methylation-Lightning kit (Zymo Research Corp, cat #D5047) on the Hamilton Star Liquid handling platform. Eight microliters of modified DNA was subjected to methylation analysis on the Illumina InfiniumMethylation EPIC BeadChip (Illumina, CA, USA) at UCL Genomics according to the manufacturer’s standard protocol.

### Methylation analysis

All methylation microarray data were processed through the same standardized pipeline. Raw data was loaded using the R package minfi, version 1.40.0 [31]. Any samples with median methylated and unmethylated intensities < 9.5 were removed. Any probes with a detection *p*-value > 0.01 were regarded as failed. Any samples with > 10% failed probes and any probes with > 10% failure rate were removed from the dataset. Beta values from failed probes (approximately 0.001% of the dataset) were imputed using the impute.knn function as part of the impute R package, version 1.68.0.

Non-CpG probes (2932), SNP-related probes as identified by Zhou et.al [32]. (82,108), and chrY probes were removed from the dataset. An additional 6102 previously identified probes that followed a trimodal methylation pattern characteristic of an underlying SNP were removed. Beta values from a total of 776,725 remained after these filtering steps.

Background intensity correction and dye bias correction was performed using the minfi single sample preprocessNoob function. Probe bias correction was performed using the beta mixture quantile normalization (BMIQ) algorithm in R package ChAMP, version 2.24.0 [33].

The fraction of immune cell contamination, and the relative proportions of different immune cell subtypes in each sample, was estimated using the EpiDISH algorithm using the epithelial, fibroblast, and immune cell reference dataset described in Zheng et al. [34]. Briefly, generic epithelial and fibroblast reference datasets were generated from eleven distinct epithelial or seven distinct fibroblast cell lines, respectively, assessed on the Illumina 450 k array as part of the ENCODE Project, while the immune cell reference dataset was generated from 42 purified samples representing all major IC types [35]. For breast tissue, the EpiDISH algorithm was run using epithelial, fibroblast, immune, and a fat cell reference dataset [34]. The EpiDISH algorithm is well-established for cell type deconvolution [36]. The top 1000 most variable probes (ranked by standard deviation) were used in a principal component analysis. Statistical tests were performed in order to identify any anomalous associations between plate, sentrix position, date of array processing, date of DNA creation, study center, immune contamination fraction, age, type (case versus control), and the top ten principal components.

### Statistical development of epigenetic clocks

The R package glmnet, version 4.1.3, with alpha = 1 (lasso penalty) was used to train a regression model to predict chronological age as the output. Beta-values from the 776,725 CpGs in the training set were used as inputs to the model. Ten-fold cross-validation was utilized to determine the optimal value of the regularization parameter lambda. The final model included non-zero weights for *n* = 759 CpG sites. Denoting the beta values as *β*_1_, …, *β*_*n*_ and the regression coefficients from the trained model as *w*_1_, …, *w*_*n*_, then the WID general clock is given ${\sum}_{i=1}^n{w}_i{\beta}_i+ intercept$, where the intercept term was also fitted during the model training phase.

In order to identify epithelial- and immune-specific age-associated changes at each CpG site, we fitted the following linear model. At a given CpG site let *β* denote the beta-value, *ic* and *ec* denote the immune and epithelial proportions, and *age* denote chronological age. We linear regressed *β* on *ic*, *ec*, *age*, *ic* ∙ *age*, and *ec* ∙ *age*. Coefficients corresponding to the interaction terms capture cell-type-specific associations between beta values and chronological age. The linear regression model was applied to all 776,725 CpGs in the training set.

Epithelial-specific CpGs were identified by selecting CpGs with an absolute epithelial-specific coefficient > 10^−3^ and an absolute immune-specific coefficient < 10^−5^ (168 in total). Similarly, immune-specific CpGs were identified by selecting CpGs with an absolute immune-specific coefficient > 10^−3^ and an absolute epithelial-specific coefficient < 10^−5^ (56 in total). The WID epithelial clock was trained on the epithelial-specific CpGs by using ridge regression to predict chronological age using *β* and *β* ∙ *ic* interaction terms as inputs. Interaction terms were included to accommodate any cell-type-specific dependencies. The final clock was defined as ${\sum}_{i=1}^{168}{w}_i^1{\beta}_i+{w}_i^2{\beta}_i{ic}_i+ intercept$. Similarly the WID immune clock was defined as ${\sum}_{i=1}^{56}{w}_i^1{\beta}_i+{w}_i^2{\beta}_i{ic}_i+ intercept$. Coefficients for all WID clocks are available in Additional files 2, 3 and 4: Supplementary Tables S7-9.

Age-acceleration was defined as the difference between the WID general clock and chronological age. Age-acceleration was adjusted for chronological *age* and *ic* by taking residuals after regressing on *age* and *ic* in the 225 control samples from Validation set 1. When analyzing HRT and OCP, we took residuals based on control samples that were never on HRT or OCP respectively. The WID-relative-epithelial-age (REA) was defined as WID epithelial clock − WID general clock, and the WID-relative-immune-age was defined as WID immune clock − WID general clock. Where indicated, both of these quantities were adjusted for *age* and *ic* based on control samples from Validation set 1. In breast tissue samples, the WID-REA was adjusted for age using the *n* = 36 normal tissue samples. In the buccal samples from Validation set 2, the WID-REA was adjusted for age and ic using samples from healthy post-menopausal controls that were never on HRT. The average immune cell composition of buccal samples was 33.9% (16.8–49%). For blood samples, adjustment was made for age only.

The odds ratio associated with breast cancer was computed by dividing the 225 control samples into WID-REA quartiles and using the oddsratio function from the epitools R package, version 0.5.10.1.

The pcgtAge mitotic clock was computed using the mean methylation across 385 promoter CpGs at polycomb group target genes. The “solo-WCGWs” mitotic clock was computed using the mean methylation across 34,276 “solo-WCGWs CpGs that are available on the EPIC array.

## Supplementary Information

**Additional file 1: Fig. S1.** Datasets and experimental design. **Fig. S2.** Detailed comparison of the WID general clock and other epigenetic clocks, and assessments of cell composition in response to menopause, hormone replacement and cancer. **Fig. S3.** Detailed epidemiological assessment of the WID general clock age acceleration. **Fig. S4.** Association of the WID general, epithelial and immune clocks with menopause, replicative age, and solo-WCGW methylation. **Fig. S5.** Association of WID-relative-epithelial- and -immune-age with chronological age and immune cell proportion and breast cancer subtype. **Fig. S6.** Epidemiological associations of the WID-relative-epithelial-age. **Fig. S7.** Epidemiological associations of the WID-relative-immune-age. **Table S1.** Epidemiological and clinical characteristics of the training set. **Table S2.** Epidemiological and clinical characteristics of the validation set 1. **Table S3.** WID-REA quantiles and pre-menopausal breast cancer risk. **Table S4.** Ages of individuals in the breast tissue sets. **Table S5.** Epidemiological and clinical characteristics of Validation set 2. **Table S6.** List of samples used to assess the performance of age prediction.

**Additional file 2: Table S7.** WID general clock intercept and coefficients.

**Additional file 3: Table S8.** WID epithelial clock intercept and coefficients.

**Additional file 4: Table S9.** WID immune clock intercept and coefficients.

**Additional file 5.** Review history.

## Data Availability

Individual datasets have been deposited in the EGA repository as part of study EGAS00001005055,EGAS00001005070 , EGAS00001005045, and EGAS00001005626. Data is available for research use only after completion of an access form and subsequent approval through the data access committee. WID clock coefficients are available in Additional files 2, 3 and 4: Supplementary Tables S7-9. The R code for calculation of the WID general, epithelial, and immune clocks is available in a github repository (https://github.com/chiaraherzog/WIDclocks) [37].
