# Author Correction: Susceptibility to hormone-mediated cancer is reflected by different tick rates of the epithelial and general epigenetic clock

**DOI:** 10.1186/s13059-022-02704-z

**Published:** 2022-06-29

**Authors:** J. James E. Barrett, Chiara Herzog, Yoo-Na Kim, Thomas E. Bartlett, Allison Jones, Iona Evans, David Cibula, Michal Zikan, Line Bjørge, Nadia Harbeck, Nicoletta Colombo, Sacha J. Howell, Angelique Flöter Rådestad, Kristina Gemzell-Danielsson, Martin Widschwendter

**Affiliations:** 1 European Translational Oncology Prevention and Screening (EUTOPS) Institute, Milser Str. 10, 6060 Hall in Tirol, Austria; 2 Research Institute for Biomedical Aging Research, Universität Innsbruck, 6020 Innsbruck, Austria; 3 Department of Statistical Science, University College London, London, WC1E 7HB UK; 4 Department of Women’s Cancer, UCL EGA Institute for Women’s Health, University College London, Medical School Building, Room 340, 74 Huntley Street, London, WC1E 6AU UK; 5 Gynaecologic Oncology Center, Department of Obstetrics and Gynecology, First Faculty of Medicine, Charles University in Prague, General University Hospital in Prague, Prague, Czech Republic; 6 Department of Gynecology and Obstetrics, Charles University in Prague, First Faculty of Medicine and University Hospital Bulovka, Prague, Czech Republic; 7 Department of Obstetrics and Gynaecology, Haukeland University Hospital, Bergen, Norway; 8 Centre for Cancer Biomarkers CCBIO, Department of Clinical Science, University of Bergen, Bergen, Norway; 9 Breast Center, Department of Obstetrics and Gynecology, University of Munich (LMU), Munich, Germany; 10 Istituto Europeo di Oncologia IRCCS, Milan, Italy; 11 University of Milano-Bicocca, Milan, Italy; 12 Breast Biology Group, Manchester Breast Centre, Division of Cancer Sciences, Faculty of Biology, Medicine and Health, University of Manchester, Manchester, UK; 13 Department of Women’s and Children’s Health, Karolinska Institutet and Karolinska University Hospital, Stockholm, Sweden

**Correction: Genome Biol 23, 52 (2022)**

**https://doi.org/10.1186/s13059-022-02603-3**

Following publication of the original article [1], it was brought to our attention that one of the references was incorrect.

The text of the article reads:

The general, epithelial and immune clocks are significantly, albeit weakly, correlated with two mitotic clocks, the pcgtAge score based on promoter CpGs at polycomb group target genes [24] and an alternative mitotic clock model recently developed using “solo-WCGWs” [25]

The correct reference for citation 24 should be as follows:

24. Yang Z, Wong A, Kuh D, Paul DS, Rakyan VK, Leslie RD et al. Correlation of an epigenetic mitotic clock with cancer risk. Genome Biology 2016; 17: 205

Also, we wish to add an additional reference to that cited as citation 25. It has been brought to our attention that it would also be relevant to cite the following:

25. Teschendorff AE. A comparison of epigenetic mitotic-like clocks for cancer risk prediction. Genome Medicine 2020; 12: 56

We apologize for the previous errors in the reference list.
