# Supplementary material for: Susceptibility to hormone-mediated cancer is reflected by different tick rates of the epithelial and general epigenetic clock
Source: Genome Biol. 2022 Feb 22;23:52. doi: 10.1186/s13059-022-02603-3 (PMC8862470; doi:10.1186/s13059-022-02603-3)
Supplement: Supplementary file 1 — Additional file 1: Fig. S1. Datasets and experimental design. Fig. S2. Detailed comparison of the WID general clock and other epigenetic clocks, and assessments of cell composition in response to menopause, hormone replacement and cancer. Fig. S3. Detailed epidemiological assessment of the WID general clock age acceleration. Fig. S4. Association of the WID general, epithelial and immune clocks with menopause, replicative age, and solo-WCGW methylation. Fig. S5. Association of WID-relative-epithelial- and -immune-age with chronological age and immune cell proportion and breast cancer subtype. Fig. S6. Epidemiological associations of the WID-relative-epithelial-age. Fig. S7. Epidemiological associations of the WID-relative-immune-age. Table S1. Epidemiological and clinical characteristics of the training set. Table S2. Epidemiological and clinical characteristics of the validation set 1. Table S3. WID-REA quantiles and pre-menopausal breast cancer risk. Table S4. Ages of individuals in the breast tissue sets. Table S5. Epidemiological and clinical characteristics of Validation set 2. Table S6. List of samples used to assess the performance of age prediction. [file 13059_2022_2603_MOESM1_ESM.pdf]

Susceptibility to hormone-mediated cancer is reflected by different tick rates of the epithelial and general epigenetic clock

Additional file 1

Contents

1 **Fig. S1. Datasets and experimental design.** **2**

2 **Fig. S2. Detailed comparison of the WID general clock and other epigenetic clocks, and assessments of cell composition in response to menopause, hormone replacement and cancer.** **3**

3 **Fig. S3. Detailed epidemiological assessment of the WID general clock age acceleration.** **3**

4 **Fig. S4. Association of the WID general, epithelial and immune clocks with menopause, replicative age, and solo-WCGW methylation.** **4**

5 **Fig. S5. Association of WID-relative-epithelial- and -immune-age with chronological age and immune cell proportion and breast cancer subtype.** **5**

6 **Fig. S6. Epidemiological associations of the WID-relative-epithelial-age.** **6**

7 **Fig. S7. Epidemiological associations of the WID-relative-immune-age.** **7**

8 **Table S1. Epidemiological and clinical characteristics of the training set.** **9**

9 **Table S2. Epidemiological and clinical characteristics of the validation set 1.** **10**

10 **Table S3. WID-REA quantiles and breast cancer risk in pre-menopausal women in validation set 1.** **11**

11 **Table S4. Ages of individuals in the breast tissue sets.** **12**

12 **Table S5. Epidemiological and clinical characteristics of validation set 2.** **13**

13 **Table S6. List of samples used to assess the performance of age prediction.** **14**

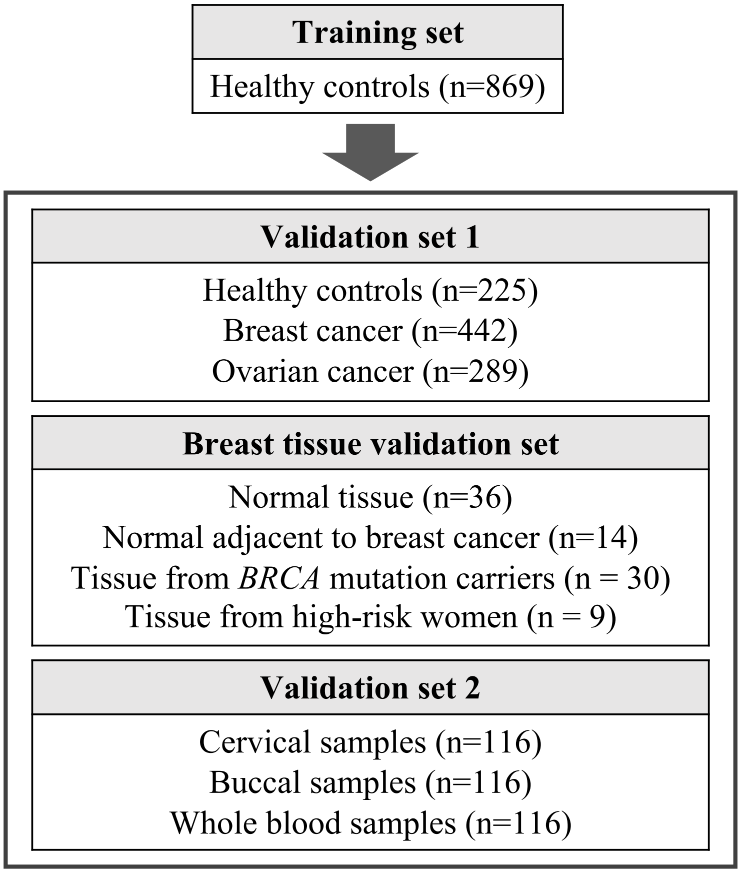

Fig. S1. Datasets and experimental design.

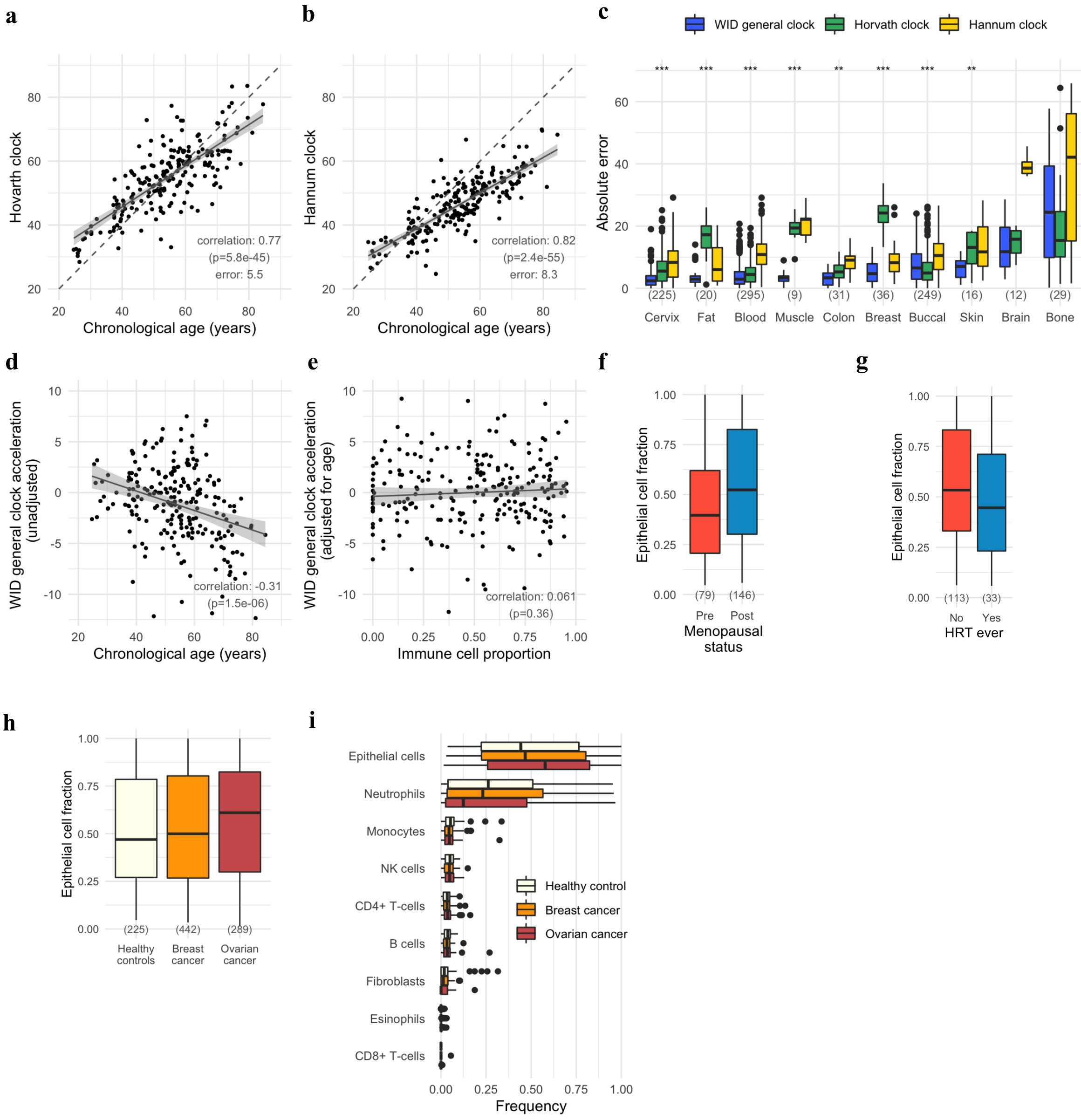

Fig. S2. Detailed comparison of the WID general clock and other epigenetic clocks, and assessments of cell composition in response to menopause, hormone replacement and cancer. Epigenetic age versus chronological age as predicted by **a** the Horvath or **b** Hannum clock. **c** Error (absolute difference between predicted and chronological age) in the WID general clock compared to existing clocks across different tissue types (p values correspond to a comparison of the WID general and Horvath clock). Age-acceleration (difference between the WID general clock and chronological age) versus **d** chronological age and **e** immune cell proportion. **f-h** show the epithelial proportion in cervical samples in response to **f** menopause, **g** HRT, or **h** in samples from different cancer patients compared to healthy controls. **i** Cell type composition in validation set 1.

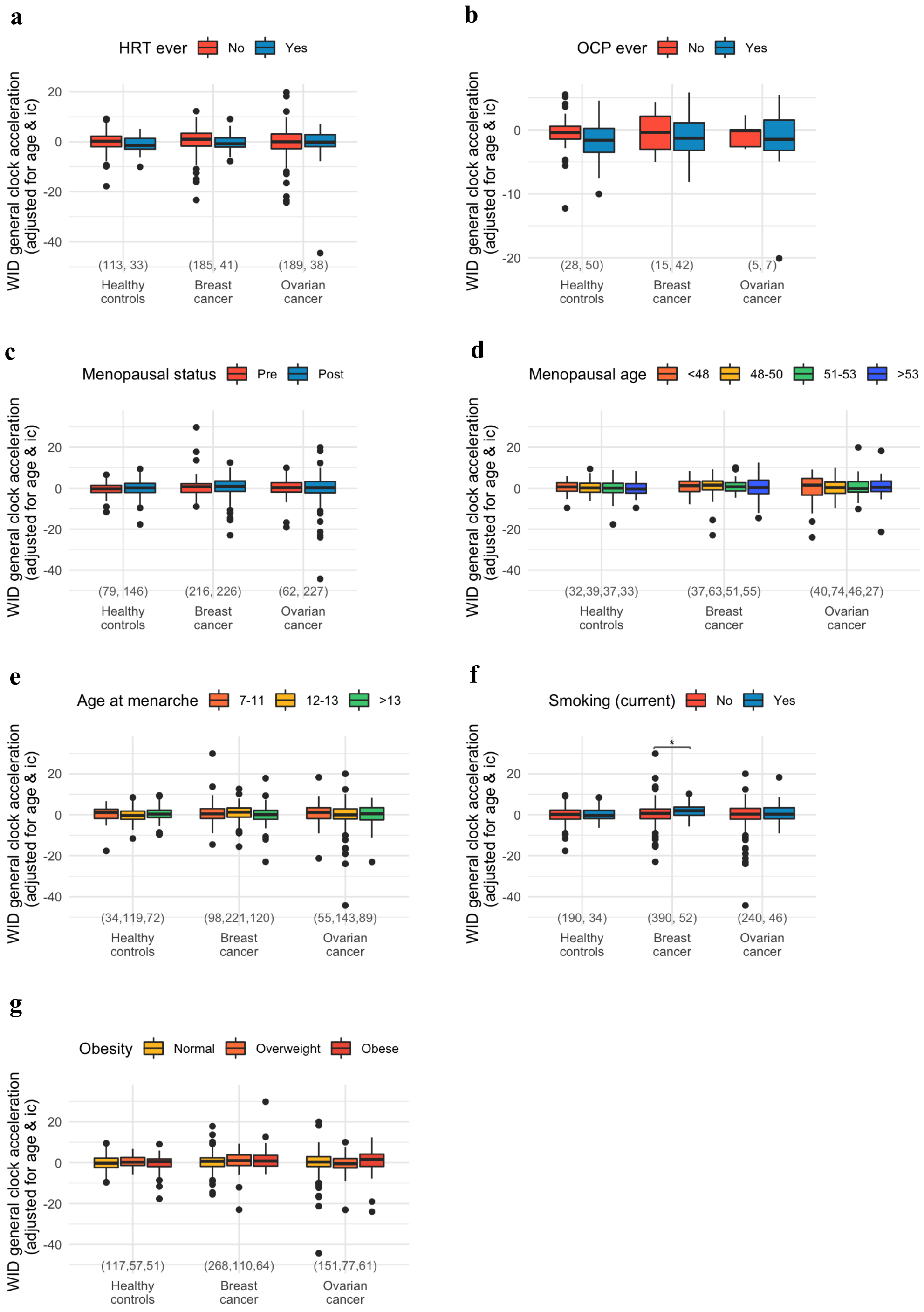

Fig. S3. Detailed epidemiological assessment of the WID general clock age acceleration. **a** The WID general clock age acceleration (adjusted for age and ic proportion) with respect to history of hormone replacement therapy (HRT) in post-menopausal women, **b** history of oral contraceptive pill (OCP) use in in pre-menopausal women, **c** menopausal status, **d** age at menopause in post-menopausal women, **e** age at menarche, **f** current smoking, and **g** obesity (normal if body mass index (BMI) <25, overweight if BMI was between 25 and 30, and obese if BMI was >30) in cervical samples from validation set 1. *, $p \leq 0.05$; ** $p \leq 0.01$ in Wilcoxon signed-rank test.

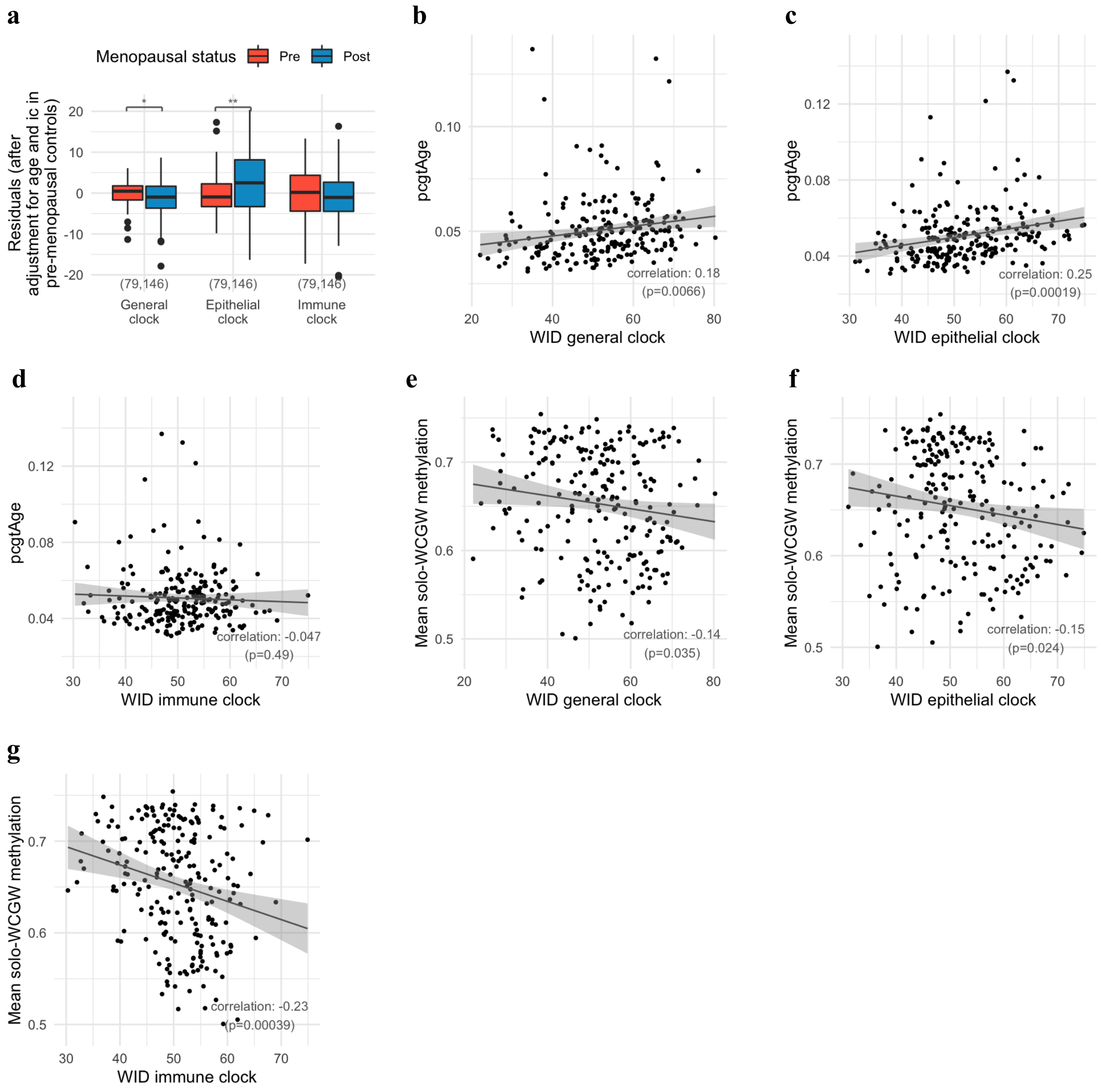

Fig. S4. Association of the WID general, epithelial and immune clocks with menopause, replicative age, and solo-WCGW methylation. **a** WID general, epithelial, and immune clocks with respect to menopausal status. **b** pcgtAge versus WID general clock, **c** WID epithelial clock, and **c** WID immune clock . **e** Mean solo-WCGW methylation (CpGs in locations of preferential hypomethylation in partially methylated domains) versus WID general clock, **f** WID epithelial clock, and **g** WID immune clock. Correlation and p values were obtained using Pearson product-moment correlation. *, p $\leq$ 0.05; **, p $\leq$ 0.01 in Wilcoxon signed-rank test.

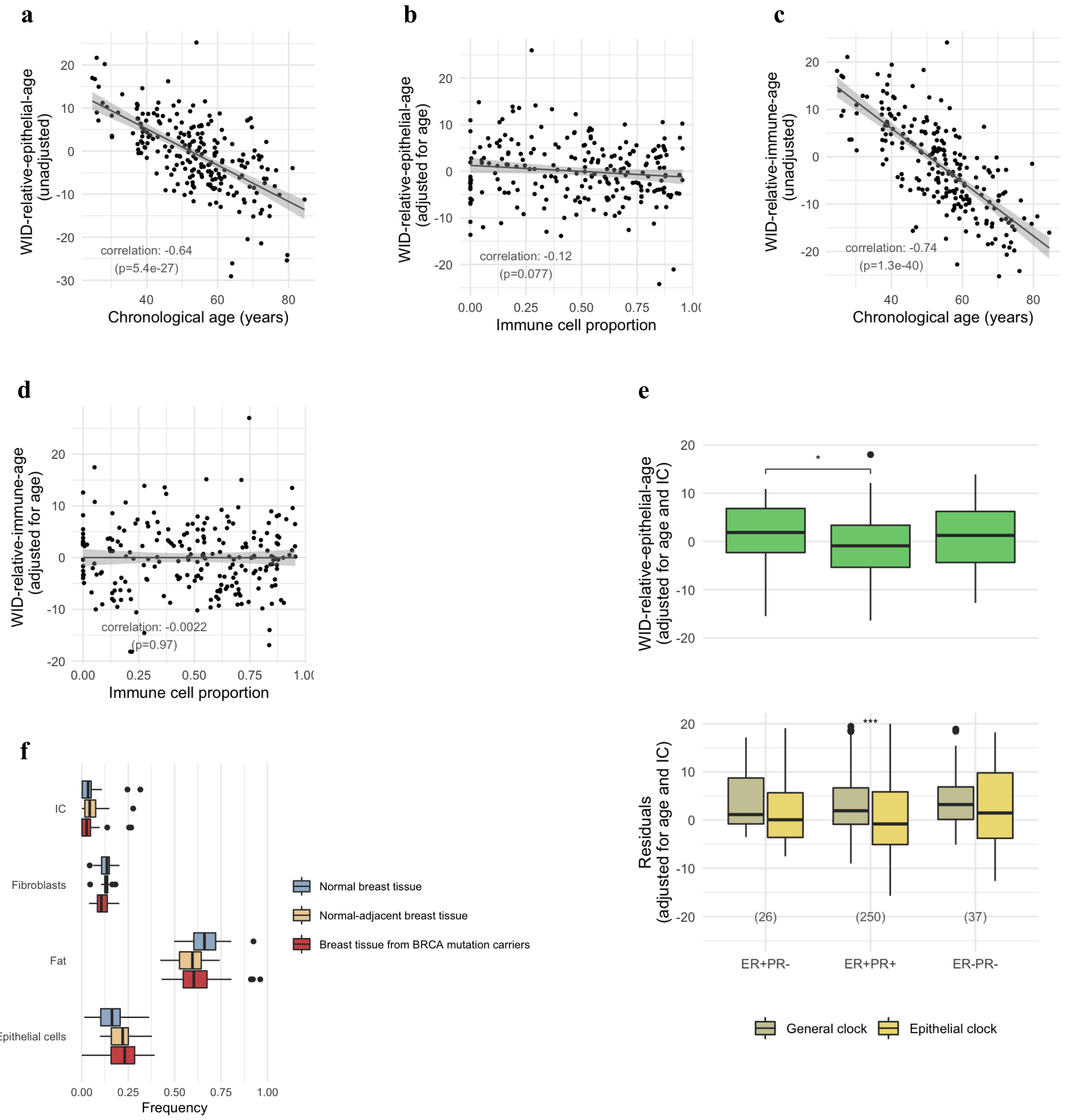

Fig. S5. Association of WID-relative-epithelial- and -immune-age with chronological age and immune cell proportion and breast cancer subtype. **a** WID-relative-epithelial-age versus chronological age and **b** ic proportion in cervical samples from controls from validation set 1. WID-relative-immune-age versus **c** chronological age and **d** ic proportion in cervical samples from controls from validation set 1. **e** WID-relative-epithelial-age, general, epithelial clocks in breast cancers. **f** Cell type composition of samples in the breast tissue set. *, $p \leq 0.05$; ** $p \leq 0.01$ in (paired or unpaired) Wilcoxon signed-rank test, respectively. Correlations and p values in **a-d** were obtained using Pearson product-moment correlation.

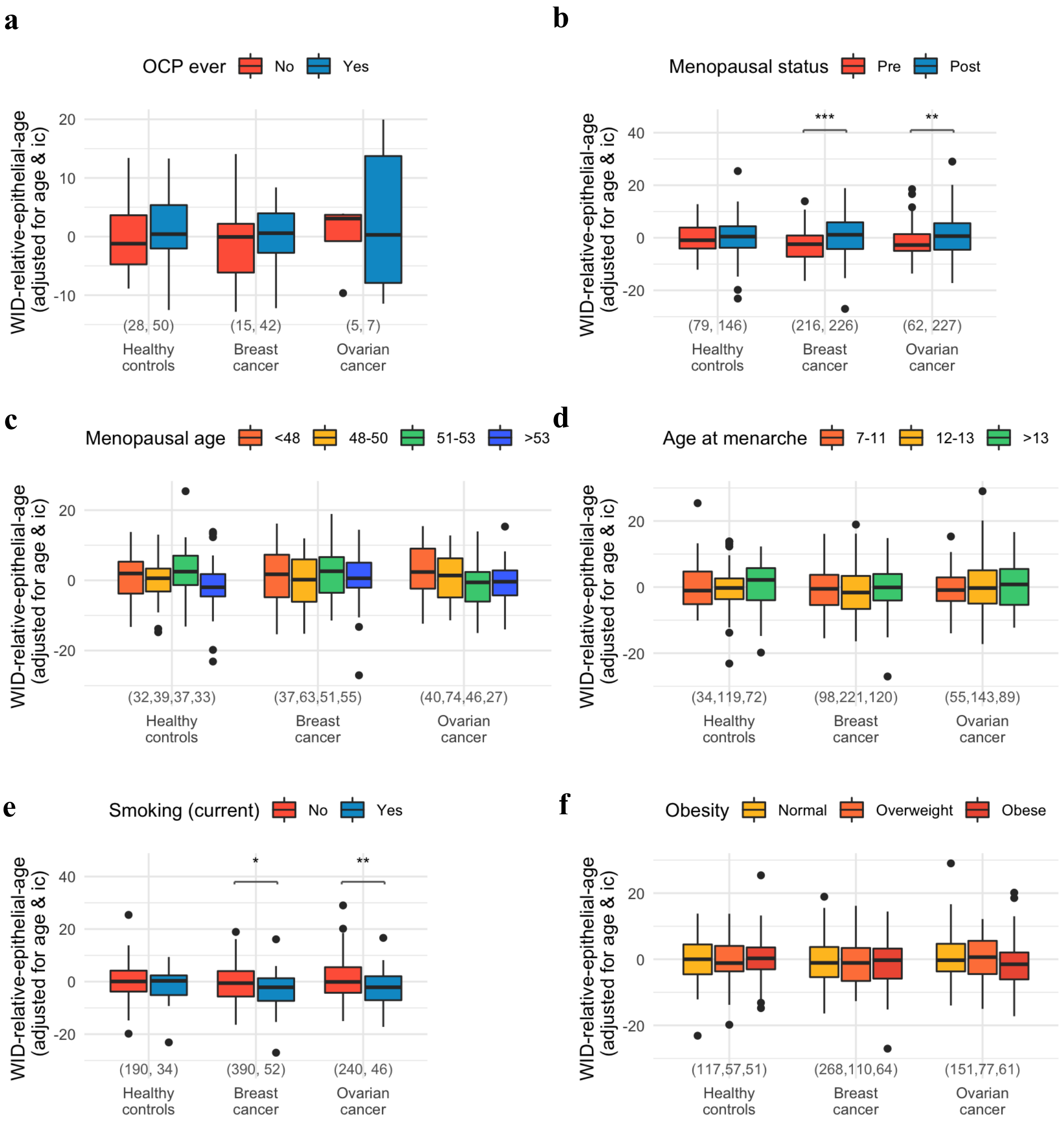

Fig. S6. Epidemiological associations of the WID-relative-epithelial-age. **a** WID-REA (adjusted for age and ic proportion) with respect to history of oral contraceptive pill (OCP) use in pre-menopausal women, **b** menopausal status, **c** age at menopause in post-menopausal women, **d** age at menarche, **e** current smoking and **f** obesity (normal if body mass index (BMI) <25, overweight if BMI was between 25 and 30, and obese if BMI was >30) in cervical samples from validation set 1. *, $p \leq 0.05$; ** $p \leq 0.01$; *** $p \leq 0.001$ in Wilcoxon signed-rank test.

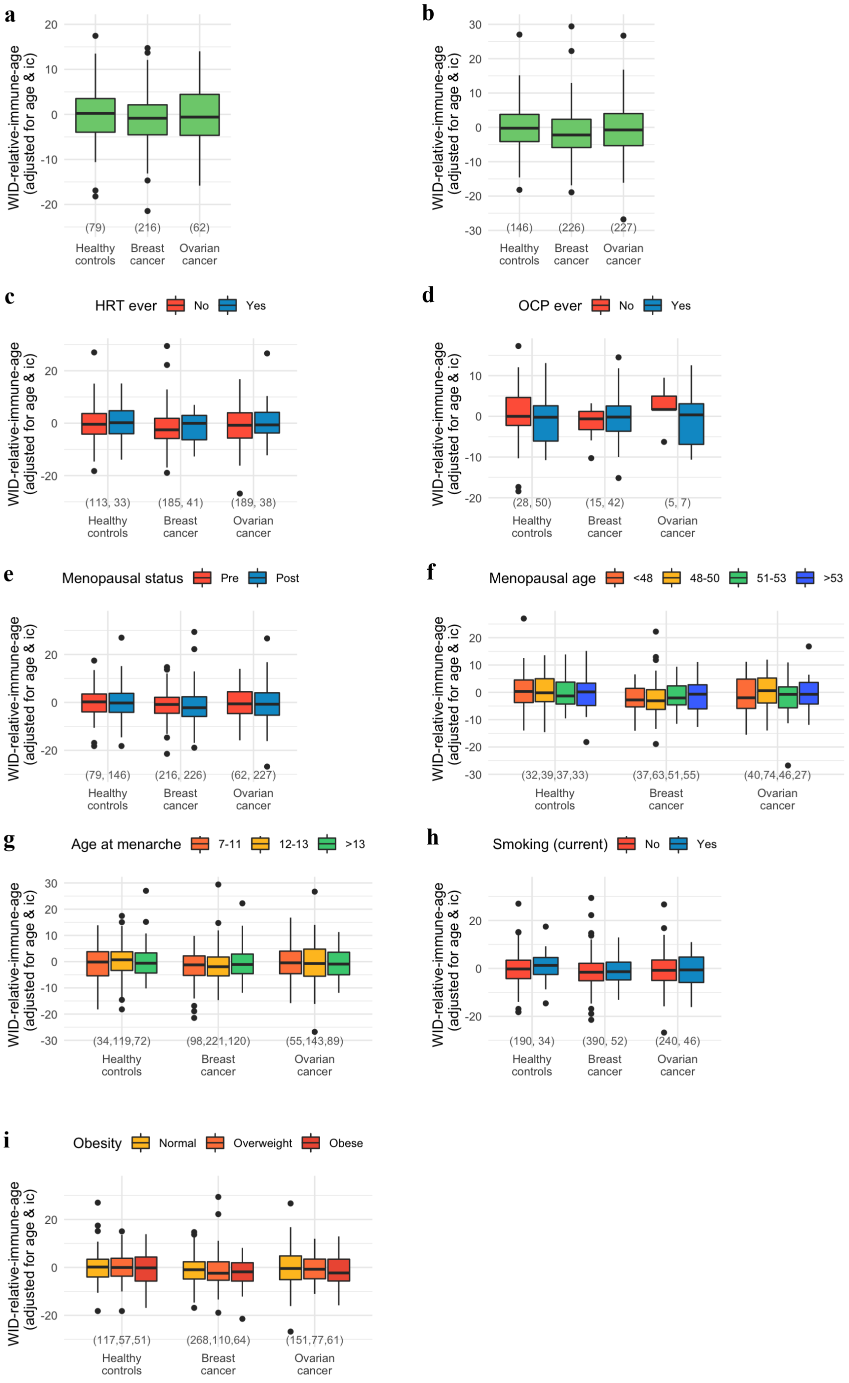

Fig. S7. Epidemiological associations of the WID-relative-immune-age. a WID-relative-immune-age (adjusted for age and ic proportion) with respect to pre-menopausal controls and cancers, **b** post-menopausal controls and cancers, **c** history of hormonal replacement therapy (HRT) in post-menopausal women, **d** history of oral contraceptive pill (OCP) use in pre-menopausal women, **e** menopausal status, **f** age at menopause in post-menopausal women, **g** age at menarche, **h** current smoking and **i** obesity (normal if body mass index (BMI) <25, overweight if BMI was between 25 and 30, and obese if BMI was >30) in samples from validation set 1. *, $p \leq 0.05$ in Wilcoxon signed-rank test.

Table S1. Epidemiological and clinical characteristics of the training set.

	Healthy controls n=869
**Age (years)**	
<52	538 (62%)
52-64	208 (24%)
>64	123 (14%)
**Menopausal status**	
Pre	509 (59%)
Post	360 (41%)
**Age at menopause (years)**	
<46	423 (49%)
46-52	260 (30%)
>52	101 (12%)
Missing or Unknown	85 (10%)
**Age at menarche (years)**	
<12	150 (17%)
12-13	437 (50%)
>13	277 (32%)
Missing or Unknown	5 (1%)
**Parous**	
Yes	636 (73%)
No	232 (27%)
Missing or Unknown	1 (0%)
**Age at birth of first live child (years)**	
<21	22 (3%)
21-27	211 (34%)
>27	396 (63%)
Missing or Unknown	240 (28%)
**Hormone replacement therapy (postmenopausal only)**	
No	247 (69%)
Yes	112 (31%)
Missing or Unknown	1 (0%)
**Current smoking**	
No	759 (87%)
Yes	95 (11%)
Missing or Unknown	15 (2%)
**BMI (kg/m^2)**	
<25	445 (51%)
25-30	275 (32%)
>30	149 (17%)

Table S2. Epidemiological and clinical characteristics of the validation set 1.

	Healthy controls n=225	Breast cancer n=442	Ovarian cancer n-289
**Age (years)**			
<52	91 (40%)	223 (50%)	77 (27%)
52-64	85 (38%)	123 (28%)	103 (36%)
>64	49 (22%)	96 (22%)	109 (38%)
**Menopausal status**			
Pre	79 (35%)	216 (49%)	62 (21%)
Post	146 (65%)	226 (51%)	227 (79%)
**Age at menopause (years)**			
<46	82 (36%)	141 (32%)	66 (23%)
46-52	91 (40%)	191 (43%)	138 (48%)
>52	47 (21%)	88 (20%)	43 (15%)
Missing or Unknown	5 (2%)	22 (5%)	42 (15%)
**Age at menarche (years)**			
<12	34 (15%)	98 (22%)	55 (19%)
Dec-13	119 (53%)	221 (50%)	143 (49%)
>13	72 (32%)	120 (27%)	89 (31%)
Missing or Unknown	0 (0%)	3 (1%)	2 (1%)
**Parous**			
Yes	166 (74%)	344 (78%)	222 (77%)
No	59 (26%)	97 (22%)	65 (22%)
Missing or Unknown	0 (0%)	1 (0%)	2 (1%)
**Age at birth of first live child (years)**			
<21	16 (7%)	11 (2%)	15 (5%)
21-27	61 (27%)	103 (23%)	127 (44%)
>27	84 (37%)	226 (51%)	78 (27%)
Missing or Unknown	64 (28%)	102 (23%)	69 (24%)
**Hormone replacement therapy (postmenopausal only)**			
No	113 (77%)	185 (82%)	189 (83%)
Yes	33 (23%)	41 (18%)	38 (17%)
Missing or Unknown	0 (0%)	0 (0%)	0 (0%)
**Oral contraceptive use (premenopausal only)**			
No	28 (35%)	15 (7%)	5 (8%)
Yes	50 (63%)	42 (19%)	7 (11%)
Missing or Unknown	1 (1%)	159 (74%)	50 (81%)
**Current smoking**			
No	190 (84%)	390 (88%)	240 (83%)
Yes	34 (15%)	52 (12%)	46 (16%)
Missing or Unknown	1 (0%)	0 (0%)	3 (1%)
**BMI (kg/m²)**			
<25	117 (52%)	268 (61%)	151 (52%)
25-30	57 (25%)	110 (25%)	77 (27%)
>30	51 (23%)	64 (14%)	61 (21%)

Table S3. WID-REA quantiles and breast cancer risk in pre-menopausal women in validation set 1. WID-REA quantiles were defined based on control samples in validation set 1. Odds ratios for quantiles were computed using the highest quantile as the reference. Odds ratios and p values were determined using median-unbiased estimation.

Quantile	Control	Cancer	Odds Ratio	95% CI	p
**(3.03 to 16.79)**	**20**	**32**	**1 (Reference)**	**-**	**-**
(-0.11 to 3.03)	20	49	1.52	0.71-3.31	0.28
(-3.31 to -0.11)	19	48	1.57	0.72-3.44	0.25
**(-16.91 to -3.31)**	**20**	**87**	**2.7**	**1.28-5.72**	**0.009**

Table S4. Ages of individuals in the breast tissue sets.

Set	Age, mean (range)
Normal breast tissue	28.97 (19-51)
Normal-adjacent breast tissue	43.14 (32-54)
Breast tissue from *BRCA1/2* mutation carriers	35.07 (22-51)

Table S5. Epidemiological and clinical characteristics of validation set 2.

	**Healthy controls**<br>**n=116**
**Age (years)**	
<52	91 (78%)
52-64	15 (13%)
>64	10 (9%)
**Menopausal status**	
Pre	91 (78%)
Post	25 (22%)
**Age at menopause (years)**	
<46	78 (67%)
46-52	28 (24%)
>52	5 (4%)
Missing or Unknown	5 (4%)
**Age at menarche (years)**	
<12	28 (24%)
12-13	55 (47%)
>13	32 (28%)
Missing or Unknown	1 (1%)
**Parous**	
Yes	51 (44%)
No	65 (56%)
**Hormone replacement therapy (postmenopausal only)**	
No	13 (52%)
Yes	12 (48%)
**Current smoking**	
No	105 (91%)
Yes	11 (9%)
**BMI (kg/m^2)**	
<25	74 (64%)
25-30	28 (24%)
>30	14 (12%)

Table S6. List of samples used to assess the performance of age prediction.

	Number of samples
**Fat**	
GSE131461	20
**Blood**	
GSE131461	20
GSE145254	23
GSE143307	48
GSE130748	19
GSE123914	69
**Muscle**	
GSE114763	9
**Colon**	
GSE142257	31
**Buccal**	
GSE157252	134
**Skin**	
GSE151617	16
**Brain**	
GSE138597	12
**Bone**	
GSE138307	29
