# Supplementary material for: Susceptibility to hormone-mediated cancer is reflected by different tick rates of the epithelial and general epigenetic clock
Source: Genome Biol. 2022 Feb 22;23:52. doi: 10.1186/s13059-022-02603-3 (PMC8862470; doi:10.1186/s13059-022-02603-3)
Supplement: Supplementary file 5 — Additional file 5. Review history. [file 13059_2022_2603_MOESM5_ESM.docx]

**1^st^ round**

**Reviewer 1**

This study follows an interesting approach to develop independent epigenetic clocks for epithelial, immune, and general cellular subsets. In fact, it is well conceivable that not all cell types within a tissue age at the same pace. A major strength of the study is the large number of cervical cytology samples, which can be an important resource to other researchers. The authors indicate that the cell-type specific epigenetic clocks might be differentially affected by menopause, hormonal treatment or cancer. These effects are overall relatively small with regard to the absolute deviation of age predictions, but they are significant given the high number of samples. I have the following specific comments:

1) The cellular composition is an important parameter for this study. Apparently, the cellular composition was estimated with EpiDISH and is not based on "real" cell counts. This method should be better clarified in the manuscript and the authors should critically discuss that their reference dataset (e.g. epithelial cells from iris) may evoke some offset.

2) The approach to develop clocks based on epithelial-specific and immune-specific CpGs is straight forward, but the authors should prove that in pure cell preparations the clocks are really only functional for the specific cell types (for example, the epithelial clock should hardly correlate with chronological age in pure datasets of immune cells).

3) On the other hand, it is well conceivable that menopause, disease, and hormone treatment affect the cellular composition in specimen. The authors should use their EpiDISH approach to investigate such potential confounders. If there are differences in the cellular composition this will notoriously affect the signatures and age-predictions, even if the CpGs are not age-associated in these cell types.

Minor:

4) Buccal samples have also a high proportion of leukocytes. This may be discussed (and determined).

5) The "immune cell proportion" might be better clarified. What immune cell types are specifically considered here?

6) The molecular clocks might be provided as supplemental table or code to be applicable by other researchers.

7) The authors compared their epigenetic clock with the clocks of Horvath and Hannum of 2013. It should be mentioned that there are several alternative signatures, e.g. the more recent skin and blood clock by Horwath (PMID: 30048243) that might perform better on this EPIC dataset.

8) The figure legends are in general very brief. It would be easier for the reader if more a more detailed description would be provided.

9) Perhaps the authors might comment briefly under what condition the raw data is (publicly) accessible on the EGA repository?

**Reviewer 2**

This is an interesting manuscript by Martin Widschwendter and colleagues in which the authors expand the concept of general epigenetic clock by developing cell type-specific (epithelial and immune) epigenetic clocks upon DNA methylation analysis of cervical cytology samples from 2.222 women. Overall, the authors major finding is that 1) the difference between the epithelial and general epigenetic clock, which they denote WID-REA, is significantly reduced in cervical samples from pre-menopausal women with breast cancer as compared to samples from heathy controls, 2) that WID-REA is associated with breast cancer risk and is affected by hormonal modulation. Interestingly, this perhaps unexpected finding points to a potential use of WID-REA as a novel biomarker for assessing breast cancer risk and for evaluation of (anti-)hormone treatment outcome, based on analysis of cervical cytology samples. This in indeed an interesting concept that will likely inspire the development and investigation of discordance between tissue-specific epigenetic clocks in other organs.

The manuscript is well written, short, and figures are well presented, especially considering that the topic and methods are sometimes complicated. In addition, the amount of work related to sample collection and methylation profiling should be complimented. Still, the many bioinformatical methods (including custom EpiDISH analysis, regression modelling, choice of data adjustment strategy, and WID-REA/residuals calculations) and shear wealth of analysis, including different patients groups/ sample types, occasionally makes the manuscript hard to overview during the first reading. Furthermore, fairly many borderline/non-significant associations are presented and samples numbers are low for some analysis. Despite this, I still fell convinced that the authors have successfully demonstrated what is indicated in their abstract, namely demonstrating that

- Cervical WID-REA is reduced in pre-menopausal breast cancer patients as compared to healthy controls (p=0.013, Figure 3A; validation set 1) and the effects seem particular pronounced for ER+PR+ cancers (figure S5A). Ideally, I would have hoped that this observation had been validated in an independent set of cervical cytology samples from breast cancer/healthy patients, but I fully acknowledge that this is a most difficult task. I agree with the author's choice to instead investigate breast tissue directly, from heathy and high risk women, as an alternative.

- WID-REA is reduced in breast tissue from BRCA mutation carriers, compared to "normal" breast tissue (Figure 1c)

- Ulipristal treatment of high risk women is associated with increase in breast tissue WID-REA (figure 1c)

- Hormone replacement therapy is associated with decrease in WID-RED in heathy post-menopausal cervical samples (validation cohort 1), but not in post-menopausal samples from cancer patients (figure 4a)

These are, at least to my understanding, the key and statistically significant observations of the paper, which I feel may merit high impact publication. Still, I have the following comments that I hope that the authors may consider:

1) Regarding statistical significance: Throughout the manuscript, p-values are given in the text and * (stars) are indicated in some figure, whereas I consider all remaining comparisons non-significant, when not otherwise indicated in the figures. However, I sometimes struggle to see exactly which statistical tests are used and which cut-off. Could the authors please better indicate which type of significance test is employed and include this information both in the text or figure legends along with the cut-off used for defining one or two * (stars) for each analysis. Maybe also a short section in the material and methods would be appropriate.

2) Regarding EpiDISH estimations: The principle of developing tissue-specific epigenetic clocks in the manuscript depends on a correct estimation of cell type composition in cervical cytology and breast tissue. Here, the authors estimate cell type composition from DNA methylation profiles using the bioinformatics EpiDISH approach and they developed a new references e.g. in order to evaluate the endothelial tumor fraction of cervical samples (fig 1g) and fat cell fraction in breast tissue?(fig S5f). I wonder if the authors are able to present data to support that the EpiDISH approach is accurate, at least for the cervical cytology samples presented: E.g. by comparing EpiDISH prediction of epithelial content to other measures (IHC, flow cytometry, QPCR other measures?) for a subset of samples? In addition, a new EpiDISH reference was built using the data sets shown in supplementary table s5. Should this list also contain fat cell datasets used for evaluation of fat cell content in breast tissue?

3) Regarding the analysis of endometrial cancer (figure 1): I appreciate the efforts of the authors to evaluate WID general de-acceleration in endometrial cancer patients in pre- and post-menopausal cervical cytology samples (fig 1f to g). However, I fell that this part of the manuscript distracts from the remaining manuscripts that focus on WID-REA in breast cancer, which is already somewhat complicated to understand. I realize that initial motivation for this study may have been age deceleration in endometrial cancer patients (given that endometrial cancer patients are referred to as "cases" in the material and methods). However, the WID general acceleration analysis in endometrial cancer patient is not mentioned in the title, abstract or discussion. I suggest to make the paper more focused on the WID-REA approach by moving the endometrial cancer results (fig. 1 e-f-g) to the supplementary material or even reserving it for a separate manuscript. I any case, the authors document a reduced general WID ticking rate in cervical cytology samples from endometrial cancers patients, which they attribute to the cancer cell content of the samples (figure 1g). Albeit interesting, this in perhaps not too surprising given the gross methylation changes and epigenetic clock alteration that are seen in many cancer types. The authors report this finding based on the analysis of 259 endometrial and 146 healthy post-menopausal samples. Yet, the authors find no effect upon analysis of a much smaller cohort of pre-menopausal endometrial cancers (22 cancers, 79 healthy). I wonder if this difference between pre- and post menopausal samples is truly related to pre/post-menopausal status or perhaps more simply that the pre-menopausal samples have low tumor DNA fraction compared to post-menopausal samples and that the result may change if more pre-menopausal samples were analyzed? In this regard, it is relatively few post-menopausal samples that have a high cancer content, and it seems that "rarer" high tumor-fraction samples may drive much of the association to WID general clock de-acceleration. Could the authors perhaps comment on this and indicate the tumor DNA fraction of the 22 pre-menopausal samples in figure 1g?

4) Regarding the tissue-specific epigenetic clocks: The WID general clock is evaluated in both validation cohort 1 (Figure 1b) and 2 (Figure S2c), and I therefore suggest to also show the performance of the WID epithelial and Immune clock in validation cohorts 2. Did I perhaps overlook this data for validation cohort 2? Also, what is the correlation/correspondence between WID epithelial and WID immune clock predictions (perhaps illustrated by using a similar plot type as figure 1b),

5) Regarding the tissue-specific epigenetic clocks in pre/post menopausal cervical samples: in line 190, the authors report that they find "no significant change in any of the clocks after menopause (fig s4a)". From figure 4a, it seems that residual are larger for Age predictions by the WID epithelial clock in 146 post- vs 79 pre-menopausal samples (Is this difference in residuals not statistically significant?). How does at similar analysis look for validation cohort 2. Would it be possible to report WID general, epithelial and immune clock performances (validation 1 and 2) separately for pre- and post-menopausal samples, in a format similar to figures 2bc, which provide correlation coefficient, p value and error, in the supplementary figures.

6) Regarding WID-REAs evaluated in cervical samples as a surrogate marker for breast cancer risk cancer: The authors suggest that "WID-REA in cervical samples acts a systemic surrogate indicating how individual women respond to hormonal modulation and, depending on the menopausal status, how this is associated with cancer predisposition" (line 299). This is indeed a very interesting possibility, albeit is requires future research as also highlighted by the authors themselves in the discussion (line 301). Given that the authors have access to matched cervical and buccal samples from 116 women in validation cohort 2, it may be interesting to compare WID-REA evaluated in cervical and buccal samples for these 116 patients, especially considering that differences in WID-REA between women with/without HRT treatment seem greater when evaluated in Buccal than cervical samples. Would results be highly correlated, but with a greater dynamic range for buccal tissue?

7) Regarding data availability: I am happy to see that DNA methylation profiles will be available via EGA. I would encourage to also include the probe id for the three epigenetic clocks, but also acknowledge that this may be difficult due to pending patenting of the approach. Perhaps the general clock could still be shared? I also encourage that the custom references (probeID) for EpiDISH prediction are included in the manuscript.

**Authors’ response**

**Reviewer #1:**

Comments: This study follows an interesting approach to develop independent epigenetic clocks for epithelial, immune, and general cellular subsets. In fact, it is well conceivable that not all cell types within a tissue age at the same pace. A major strength of the study is the large number of cervical cytology samples, which can be an important resource to other researchers. The authors indicate that the cell-type specific epigenetic clocks might be differentially affected by menopause, hormonal treatment or cancer. These effects are overall relatively small with regard to the absolute deviation of age predictions, but they are significant given the high number of samples. I have the following specific comments:

Response: We very much thank this reviewer for these encouraging statements.

Comments: 1)	The cellular composition is an important parameter for this study. Apparently, the cellular composition was estimated with EpiDISH and is not based on "real" cell counts. This method should be better clarified in the manuscript and the authors should critically discuss that their reference dataset (e.g. epithelial cells from iris) may evoke some offset.

Response: We agree with the reviewer that it would have been preferable to assess cervical epithelial cells or cervical immune cells only, but unfortunately in practice this is not feasible due to the large amount of cell detritus which is carried over and assessed in the bulk DNA sample. Nonetheless, the EpiDISH algorithm and the principle of this is well established and we have recently tested the algorithm in purified cervical cancer cell line (HeLa) and T cells from culture which again revealed high accuracy of EpiDISH, please see below. We have further clarified the method (lines 408-420).

Comments: 2)	The approach to develop clocks based on epithelial-specific and immune-specific CpGs is straight forward, but the authors should prove that in pure cell preparations the clocks are really only functional for the specific cell types (for example, the epithelial clock should hardly correlate with chronological age in pure datasets of immune cells).

Response: In order to address this comment we have assessed the epithelial age index in (i) those cervical samples which have the highest proportion of epithelial cells (i.e. immune cell proportion <10%) and (ii) in peripheral blood samples (which almost exclusively consists of immune cells). Although there is a correlation of the epithelial age index with the chronological age in both sample types the age range of the epithelial age in peripheral blood samples is very narrow (i.e. massive overestimation of the age in young and underestimation in older women). In the plot below the correlation values were generated as follows: correlation and p values were generated using Pearson’s product-moment correlation coefficient, and the error represents the median absolute difference between the predicted age and the chronological age.

Comments: 3)	On the other hand, it is well conceivable that menopause, disease, and hormone treatment affect the cellular composition in specimen. The authors should use their EpiDISH approach to investigate such potential confounders. If there are differences in the cellular composition this will notoriously affect the signatures and age-predictions, even if the CpGs are not age-associated in these cell types.

Response: We have now added additional plots to Suppl. Fig 2 indicating the epithelial cell fraction (1-immune cell) in the following groups: pre/post menopause, HRT, and the different cancer types (breast, ovarian, endometrial) versus controls. These indicate that there is generally large variability within groups and do not highlight any major cell changes in response to HRT, cancer type or in cervical samples from different cancer types (copied items below just for illustration). Moreover, our analyses were conducted after correction for age and immune cell proportion and hence should not rely on changes in cell type composition.

Minor:

Comments: 4)	Buccal samples have also a high proportion of leukocytes. This may be discussed (and determined).

Response: We observe an average immune cell proportion of 33.9% (16.8-49%) in buccal cells and have added these numbers to the manuscript in the methods section (line 456-457).

Comments: 5)	The "immune cell proportion" might be better clarified. What immune cell types are specifically considered here?

Response: All main classes of immune cells are considered here (for details please see Supplementary Figure S2f).

Comments: 6)	The molecular clocks might be provided as supplemental table or code to be applicable by other researchers.

Response: We have now provided tables including the intercept and coefficients for the general, epithelial and immune WID clocks (Suppl Tables S7-9) as well as the code for calculation using beta values, age, and immune cell as input and providing WID general, epithelial and immune clock as output for easy use of the WID clocks by other researchers: <https://github.com/chiaraherzog/WID-clocks> (information added to manuscript on line 493-495).

Comments: 7)	The authors compared their epigenetic clock with the clocks of Horvath and Hannum of 2013. It should be mentioned that there are several alternative signatures, e.g. the more recent skin and blood clock by Horwath (PMID: 30048243) that might perform better on this EPIC dataset.

Response: We have made a reference that we have only selected these signatures because they are the ones which are quoted most frequently.

Comments: 8)	The figure legends are in general very brief. It would be easier for the reader if more a more detailed description would be provided.

Response: We have now attempted to provide more detailed descriptions of the figures where applicable, including details on statistical testing and a title for each figure.

Comments: 9)	Perhaps the authors might comment briefly under what condition the raw data is (publicly) accessible on the EGA repository?

Response: We have now included more information (490-495). Briefly, data will be available for research use only. Users will contact the data access committee and will need to complete an access request form.

**Reviewer #2:**

Comments: This is an interesting manuscript by Martin Widschwendter and colleagues in which the authors expand the concept of general epigenetic clock by developing cell type-specific (epithelial and immune) epigenetic clocks upon DNA methylation analysis of cervical cytology samples from 2.222 women. Overall, the authors major finding is that 1) the difference between the epithelial and general epigenetic clock, which they denote WID-REA, is significantly reduced in cervical samples from pre-menopausal women with breast cancer as compared to samples from heathy controls, 2) that WID-REA is associated with breast cancer risk and is affected by hormonal modulation. Interestingly, this perhaps unexpected finding points to a potential use of WID-REA as a novel biomarker for assessing breast cancer risk and for evaluation of (anti-)hormone treatment outcome, based on analysis of cervical cytology samples. This in indeed an interesting concept that will likely inspire the development and investigation of discordance between tissue-specific epigenetic clocks in other organs.

Response: We very much appreciate the encouraging statements by this reviewer.

Comments: The manuscript is well written, short, and figures are well presented, especially considering that the topic and methods are sometimes complicated. In addition, the amount of work related to sample collection and methylation profiling should be complimented. Still, the many bioinformatical methods (including custom EpiDISH analysis, regression modelling, choice of data adjustment strategy, and WID-REA/residuals calculations) and shear wealth of analysis, including different patients groups/ sample types, occasionally makes the manuscript hard to overview during the first reading. Furthermore, fairly many borderline/non-significant associations are presented and samples numbers are low for some analysis. Despite this, I still fell convinced that the authors have successfully demonstrated what is indicated in their abstract, namely demonstrating that

- Cervical WID-REA is reduced in pre-menopausal breast cancer patients as compared to healthy controls (p=0.013, Figure 3A; validation set 1) and the effects seem particular pronounced for ER+PR+ cancers (figure S5A). Ideally, I would have hoped that this observation had been validated in an independent set of cervical cytology samples from breast cancer/healthy patients, but I fully acknowledge that this is a most difficult task. I agree with the author's choice to instead investigate breast tissue directly, from heathy and high risk women, as an alternative.

- WID-REA is reduced in breast tissue from BRCA mutation carriers, compared to "normal" breast tissue (Figure 1c)

- Ulipristal treatment of high risk women is associated with increase in breast tissue WID-REA (figure 1c)

- Hormone replacement therapy is associated with decrease in WID-RED in heathy post-menopausal cervical samples (validation cohort 1), but not in post-menopausal samples from cancer patients (figure 4a)

These are, at least to my understanding, the key and statistically significant observations of the paper, which I feel may merit high impact publication.

Response: We very much appreciate this reviewer’s clear comprehension of our findings.

Still, I have the following comments that I hope that the authors may consider:

Comments: 1)	Regarding statistical significance: Throughout the manuscript, p-values are given in the text and * (stars) are indicated in some figure, whereas I consider all remaining comparisons non-significant, when not otherwise indicated in the figures. However, I sometimes struggle to see exactly which statistical tests are used and which cut-off. Could the authors please better indicate which type of significance test is employed and include this information both in the text or figure legends along with the cut-off used for defining one or two * (stars) for each analysis. Maybe also a short section in the material and methods would be appropriate.

Response: We agree with the reviewer that the information regarding different statistical tests and significance reporting were not completely clear and we have now added information on the statistical tests used and cut-offs presented in each figure legend.

Comments: 2)	Regarding EpiDISH estimations: The principle of developing tissue-specific epigenetic clocks in the manuscript depends on a correct estimation of cell type composition in cervical cytology and breast tissue. Here, the authors estimate cell type composition from DNA methylation profiles using the bioinformatics EpiDISH approach and they developed a new references e.g. in order to evaluate the endothelial tumor fraction of cervical samples (fig 1g) and fat cell fraction in breast tissue?(fig S5f). I wonder if the authors are able to present data to support that the EpiDISH approach is accurate, at least for the cervical cytology samples presented: E.g. by comparing EpiDISH prediction of epithelial content to other measures (IHC, flow cytometry, QPCR other measures?) for a subset of samples? In addition, a new EpiDISH reference was built using the data sets shown in supplementary table s5. Should this list also contain fat cell datasets used for evaluation of fat cell content in breast tissue?

Response: While we agree that it would have been useful to further assess cell type composition using additional methods or even assess purified cells only, this would not have been practically feasible especially in cervical samples due to large amounts of debris. However, the EpiDISH algorithm is generally well established for estimation of cell type proportions in bulk tissue. We have now described the EpiDISH method more clearly on lines 408-420. Moreover, we have recently further addressed the issue of validating the performance of the EpiDISH algorithm in cervical cell samples and processed pure HeLa or pure Jurkat T cell lines which revealed high accuracy of the EpiDISH algorithm (0% inferred immune cell proportion for epithelial HeLa cell line, ~ 100% immune cell proportion inferred for immune cell line).

For the amended EpiDISH algorithm in breast cells, the reviewer is correct and we have indeed included fat cells in the reference dataset (reference generation described by Zheng et al., 2018) as well as updated this in Fig. S5. We have added this information to our methods section (line 414-415).

Comments: 3)	Regarding the analysis of endometrial cancer (figure 1): I appreciate the efforts of the authors to evaluate WID general de-acceleration in endometrial cancer patients in pre- and post-menopausal cervical cytology samples (fig 1f to g). However, I fell that this part of the manuscript distracts from the remaining manuscripts that focus on WID-REA in breast cancer, which is already somewhat complicated to understand. I realize that initial motivation for this study may have been age deceleration in endometrial cancer patients (given that endometrial cancer patients are referred to as "cases" in the material and methods). However, the WID general acceleration analysis in endometrial cancer patient is not mentioned in the title, abstract or discussion. I suggest to make the paper more focused on the WID-REA approach by moving the endometrial cancer results (fig. 1 e-f-g) to the supplementary material or even reserving it for a separate manuscript. I any case, the authors document a reduced general WID ticking rate in cervical cytology samples from endometrial cancers patients, which they attribute to the cancer cell content of the samples (figure 1g). Albeit interesting, this in perhaps not too surprising given the gross methylation changes and epigenetic clock alteration that are seen in many cancer types. The authors report this finding based on the analysis of 259 endometrial and 146 healthy post-menopausal samples. Yet, the authors find no effect upon analysis of a much smaller cohort of pre-menopausal endometrial cancers (22 cancers, 79 healthy). I wonder if this difference between pre- and post menopausal samples is truly related to pre/post-menopausal status or perhaps more simply that the pre-menopausal samples have low tumor DNA fraction compared to post-menopausal samples and that the result may change if more pre-menopausal samples were analyzed? In this regard, it is relatively few post-menopausal samples that have a high cancer content, and it seems that "rarer" high tumor-fraction samples may drive much of the association to WID general clock de-acceleration. Could the authors perhaps comment on this and indicate the tumor DNA fraction of the 22 pre-menopausal samples in figure 1g?

Response: We agree with the reviewer that the addition of the endometrial cancer data rendered the story more complex and we have now decided to remove this data from the manuscript to include it in a separate publication in order to make this manuscript more focused on the WID-REA in breast (and ovarian) cancer. As such, we have removed endometrial cancer samples throughout the manuscript.

Comments: 4)	Regarding the tissue-specific epigenetic clocks: The WID general clock is evaluated in both validation cohort 1 (Figure 1b) and 2 (Figure S2c), and I therefore suggest to also show the performance of the WID epithelial and Immune clock in validation cohorts 2. Did I perhaps overlook this data for validation cohort 2? Also, what is the correlation/correspondence between WID epithelial and WID immune clock predictions (perhaps illustrated by using a similar plot type as figure 1b),

Response: We have now assessed the data in validation set 2 (see below). Of note, women in this set were mainly premenopausal. Correlation and p values were generated using Pearson’s product-moment correlation coefficient, and error represents the median absolute difference between the predicted age and the chronological age. The epithelial age index is in fact a cervical epithelial index and hence correlates less well with chronological age in buccal epithelial samples. It is important to keep in mind that in our manuscript we are assessing the relative (and not the absolute) epithelial age for our risk predictive purposes.

Comments: 5)	Regarding the tissue-specific epigenetic clocks in pre/post menopausal cervical samples: in line 190, the authors report that they find "no significant change in any of the clocks after menopause (fig s4a)". From figure 4a, it seems that residual are larger for Age predictions by the WID epithelial clock in 146 post- vs 79 pre-menopausal samples (Is this difference in residuals not statistically significant?). How does at similar analysis look for validation cohort 2. Would it be possible to report WID general, epithelial and immune clock performances (validation 1 and 2) separately for pre- and post-menopausal samples, in a format similar to figures 2bc, which provide correlation coefficient, p value and error, in the supplementary figures.

Response: We agree with the reviewer that it would have been useful to look at menopause in validation set 2, unfortunately the number of post-menopausal participants in validation cohort was small and hence we did not carry out any comparison here or provide similar plots for Validation Set 2.

Comments: 6)	Regarding WID-REAs evaluated in cervical samples as a surrogate marker for breast cancer risk cancer: The authors suggest that "WID-REA in cervical samples acts a systemic surrogate indicating how individual women respond to hormonal modulation and, depending on the menopausal status, how this is associated with cancer predisposition" (line 299). This is indeed a very interesting possibility, albeit is requires future research as also highlighted by the authors themselves in the discussion (line 301). Given that the authors have access to matched cervical and buccal samples from 116 women in validation cohort 2, it may be interesting to compare WID-REA evaluated in cervical and buccal samples for these 116 patients, especially considering that differences in WID-REA between women with/without HRT treatment seem greater when evaluated in Buccal than cervical samples. Would results be highly correlated, but with a greater dynamic range for buccal tissue?

Response: We thank the reviewer for pointing out this interesting question. We have checked the correlation between age- and ic-adjusted WID-REA from buccal and cervical samples the same individual. Interestingly, the correlation overall appears rather low (see A below). The data appears to be split into four different quadrants, i.e. women with a high REA in both groups, women with a low REA in both groups, and those were REA in cervical and buccal cells appear orthogonal to each other. We have not yet found the reason for this (checking associations with menopause B) and HRT (C) revealed no clear grouping) and we will further investigate this in the future. We agree with the reviewers that the paper is already rather complex and hence we’d prefer not to add these data to the manuscript.

Comments: 7)	Regarding data availability: I am happy to see that DNA methylation profiles will be available via EGA. I would encourage to also include the probe id for the three epigenetic clocks, but also acknowledge that this may be difficult due to pending patenting of the approach. Perhaps the general clock could still be shared? I also encourage that the custom references (probeID) for EpiDISH prediction are included in the manuscript.

Response: We have now provided tables for the overall WID-age index and the immune and epithelial-specific age indices. We have also provided short scripts that should enable easier application of these indices for other researchers.

**2^nd^ round**

**Reviewer 1**

The authors have convincingly addressed each of my comments. Personally, I would have hoped that the issue on the cellular composition (comment 1) was discussed more critically also in the discussion. I understand that it is not trivial to isolate cervical epithelial cells, but as indicated in comment 4 to reviewer 2 there are clear epigenetic differences in different epithelial cell types. The reply to my comment 2 might also be integrated into the manuscript. The epithelial clock does have a moderate correlation with age in blood, indicating that the tissue-specific clocks are not entirely independent. Overall, the study comprises a remarkable datasets and it has several innovative findings that will be of relevance to the field.

**Reviewer 2**

I want to congratulate the authors for the improvements made to their manuscript. In particular, I find the manuscript easier to read after omitting the section on endometrial cancer. I have only few additional comments Regarding comment 1: I appreciate the included details regarding statistical testing. What does bracket indicate e.g. [*] in figure 3b indicate? Regarding comment 2: Regarding EpiDISH, I agree with the authors that evaluation of cell type composition in cervical samples may be difficult to perform by alternative methods and that the current application the EpiDISH method is sufficient. Regarding comment 3: The manuscript is now much easier to read. Regarding comment 4: Would it not be informative to readers to include the cervical WID general, epithelial and immune clock performance for validation cohort 2 the figures presented in the reviewer response only) either in figure 2 or in the supplementary material, as these represent completely independent samples? Regarding comment 5: I agree that the low content of post-menopausal samples in validation set 2 may be insufficient to provide the requested plot. Still, could menopause status not be indicated in figure 2b e.g. using e.g. circles and triangles? Also, are the residuals statically significant larger for age predictions by the WID epithelial clock in 146 post- vs 79 pre-menopausal samples in figure S4a, while not different for the WID general and Immune clock? Regarding comment 6: I agree that the additional figures presented in the reviewer response should not be included in the manuscript. Maybe these new observations could be mentioned briefly in the discussion? Regarding comment 7: I appreciate the addition of indices and scripts.
